# Effects of a novel mobile health intervention compared to a multi-component behaviour changing program on body mass index, physical capacities and stress parameters in adolescents with obesity: a randomized controlled trial

**DOI:** 10.1186/s12887-021-02781-2

**Published:** 2021-07-09

**Authors:** A. Stasinaki, D. Büchter, C.-H. I. Shih, K. Heldt, S. Güsewell, B. Brogle, N. Farpour-Lambert, T. Kowatsch, D. l’Allemand

**Affiliations:** 1 Pediatric Endocrinology, Children’s Hospital of Eastern Switzerland, St. Gallen, Switzerland; 2 Adolescent Medicine, Children’s Hospital of Eastern Switzerland, St. Gallen, Switzerland; 3 Centre for Digital Health Interventions, Department of Management, Technology, and Economics, ETH Zurich, Zurich, Switzerland; 4 Clinical Trials Unit, Cantonal Hospital of St. Gallen, St. Gallen, Switzerland; 5 Service of Endocrinology, Diabetology, Nutrition and Therapeutic Patient Education, Department of Medicine, University Hospitals of Geneva, Geneva, Switzerland; 6 Child and Youth School Health Service, Department of Education and Youth, Geneva, Switzerland; 7 Centre for Digital Health Interventions, Institute of Technology Management, University of St.Gallen, St. Gallen, Switzerland; 8 Saw Swee Hock School of Public Health, National University of Singapore, Singapore, Singapore

**Keywords:** Obesity, Adolescents, Digital health interventions, Conversational agent, Fitness, Biofeedback, Stress

## Abstract

**Background:**

Less than 2% of overweight children and adolescents in Switzerland can participate in multi-component behaviour changing interventions (BCI), due to costs and lack of time. Stress often hinders positive health outcomes in youth with obesity. Digital health interventions, with fewer on-site visits, promise health care access in remote regions; however, evidence for their effectiveness is scarce.

**Methods:**

This randomized controlled not blinded trial (1:1) was conducted in a childhood obesity center in Switzerland. Forty-one youth aged 10–18 years with body mass index (BMI) > P.90 with risk factors or co-morbidities or BMI > P.97 were recruited. During 5.5 months, the PathMate2 group (PM) received daily conversational agent counselling via mobile app, combined with standardized counselling (4 on-site visits). Controls (CON) participated in a BCI (7 on-site visits). We compared the outcomes of both groups after 5.5 (T1) and 12 (T2) months. Primary outcome was reduction in BMI-SDS (BMI standard deviation score: BMI adjusted for age and sex). Secondary outcomes were changes in body fat and muscle mass (bioelectrical impedance analysis), waist-to-height ratio, physical capacities (modified Dordel-Koch-Test), blood pressure and pulse. Additionally, we hypothesized that less stressed children would lose more weight. Thus, children performed biofeedback relaxation exercises while stress parameters (plasma cortisol, stress questionnaires) were evaluated.

**Results:**

At intervention start median BMI-SDS of all patients (18 PM, 13 CON) was 2.61 (obesity > + 2SD). BMI-SDS decreased significantly in CON at T1, but not at T2, and did not decrease in PM during the study. Muscle mass, strength and agility improved significantly in both groups at T2; only PM reduced significantly their body fat at T1 and T2. Average daily PM app usage rate was 71.5%. Cortisol serum levels decreased significantly after biofeedback but with no association between stress parameters and BMI-SDS. No side effects were observed.

**Conclusions:**

Equally to BCI, PathMate2 intervention resulted in significant and lasting improvements of physical capacities and body composition, but not in sustained BMI-SDS decrease. This youth-appealing mobile health intervention provides an interesting approach for youth with obesity who have limited access to health care. Biofeedback reduces acute stress and could be an innovative adjunct to usual care.

**Supplementary Information:**

The online version contains supplementary material available at 10.1186/s12887-021-02781-2.

## Background

Children and adolescents with overweight and obesity are a global health issue. Prevention is recognized as the most efficient means of curbing the epidemic and interventions that focus on physical activity alone or combined with a dietary intervention have been shown to reduce the risk of obesity in children and adolescents aged 6 to 18 years in a prevention setting [1]. However, many children already require urgent medical care due to severe orthopaedic, cardiovascular, metabolic and psychological co-morbidities during growth, with increased risk of premature mortality in adulthood [2].

Unlike for other common paediatric diseases, there is no “silver bullet” in the management of obesity; it requires time and intensive effort by health care professionals (HCP) and parents to understand the underlying factors and agree on a plan of action. The evidence shows that multi-component behaviour changing interventions (BCI) involving diet, physical activity and behaviour modification may be beneficial in achieving small, short-term reductions in body mass index (BMI), BMI z-score (BMI adjusted for age and sex and also called BMI standard deviation score [BMI-SDS]), as well as weight in children aged 6 to 11 years, with a very low occurrence of adverse events [3]. In adolescents with overweight or obesity, BCI can reduce measures of BMI or weight, respectively, as long as for 18 to 24 months, with low to moderate quality evidence, with, however, unclear rate of adverse events [3].

In Switzerland, a small number of specialized centres for childhood obesity have been developed but are failing to reach all the patients in need. Approximately 120,000 children and adolescents with overweight or obesity need medical care due to the severity of the disease and/or obesity-related complications. In 2008, a structured multi-professional BCI in group setting, was developed and reimbursed by health insurances [4, 5]. Unfortunately, less than 2% of patients were able to participate in group programs due to limited personal and financial resources, and the distance from specialised centre [6]. At the Children’s Hospital of Eastern Switzerland, we also developed an individual multi-component BCI and showed similar beneficial effects [7]. However, both types of BCI (individual or in group setting) are expensive and time-consuming for HCP, patients and their families.

Digital health interventions could offer a cost- and time-efficient solution, but although the number of digital interventions for treating obesity in adolescents is increasing [8], there is a paucity of evidence on health improvement [9]. Therefore, the objective of the current study was to assess a novel obesity management that moves the focus from on-site consultations in a specialized childhood obesity center to an appealing youth-friendly low-threshold mobile intervention (PathMate2), under the supervision of pediatric obesity experts. PathMate2 (PM) uses conversational agent counselling via a mobile app to support adolescents with overweight or obesity in adopting a healthy lifestyle. Specifically, it encourages them to exercise regularly, have a balanced diet and reduce, through breathing exercises, emotional stress [10] and/or loss of impulse control, which can lead to overweight [11]. Our main hypothesis was that the PM intervention would result in a reduction in BMI-SDS after 1 year, compared to a control group (CON) receiving the usual standardized multi-component BCI. We also tested whether changes in body composition, waist-to-height ratio, physical performance, blood pressure, heart rate and stress parameters differed significantly between the groups. We finally explored whether patient’s or family’s characteristics were related to changes in BMI-SDS or whether greater BMI-SDS reduction could be predicted by higher levels of physical performance and lower levels of stress achieved through biofeedback relaxation exercises [12, 13].

## Methods

### Design and study population

A simple randomized (1:1) controlled not blinded trial was conducted in the Children’s Hospital of Eastern Switzerland, which is a specialised childhood obesity management center (trial registration number: NCT03270423, date of registration: 26/07/2017, www.clinicaltrials.gov). Between September 2016 and September 2017, we recruited study participants among adolescents with overweight or obesity aged 10 to 18 years, requesting treatment in our center. Subjects were either referred by their physician or contacted us after seeing the study advertisement (flyers, media release). Participants were eligible if they had a BMI above the 90th percentile (>P.90) [14] with at least one risk factor or co-morbidity or a BMI above the 97th percentile (>P.97). Exclusion criteria were: major somatic or psychiatric diseases, assessed as described elsewhere [15], without adequate treatment; taking medication with the potential to affect weight (e.g. antiepileptic drugs or methylphenidate); participation in another obesity treatment program in the past year or already using a mobile phone application which promotes weight loss. The study took place between January 2017 and November 2018 according to study protocol. It included an intensive intervention phase of 5.5 months and a maintenance phase of 6 months. Study physicians recruited 41 study participants who, under the surveillance of the independent hospital pharmacist, were randomized to either the PM group or the CON group using the “randList” software (datinf gmbh Tübingen). To motivate children and adolescents to participate in the study, we offered five smartphones per group to the five most “successful” participants of the PM and the CON group according to a score of maximum 15 points, taking into account participation (missing appointments resulted in fewer points) and changes in physical performance and BMI-SDS.

### Intensive intervention phase

Baseline investigations were performed, on average, 5 weeks before the start of the intervention. After baseline investigations and randomization, the intensive intervention phase started, lasting 5.5. months and followed by two visits (at nine and 12 months after intervention start) to ensure maintenance of behavioural changes (maintenance phase, see below).

**Fig. 1:**
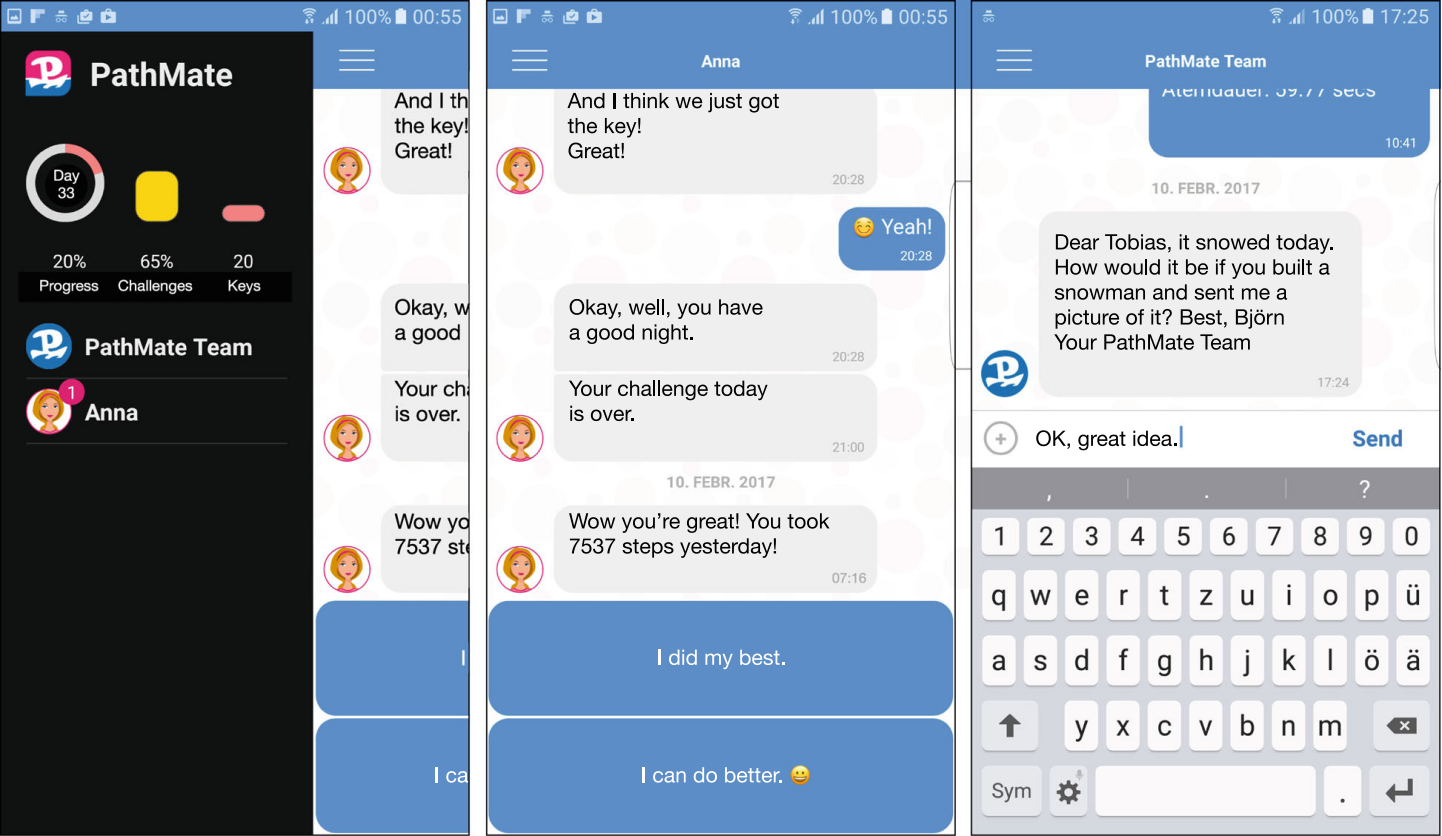
Screenshots of the PathMate2 app. Legend Fig. 1: Side menu with the dashboard (indicating the progress, the percentage of challenges completed, and the number of gamified keys that were provided for each successful challenge), chat channels for the therapeutic PathMate2 team (human coaches) and the conversational agent Anna (left screenshot), chat interaction with Anna and pre-defined answer options (middle screenshot), and chat interaction with the human coach Björn (right screenshot)

During the intensive intervention phase, the PM group had an individual multi-component BCI following Swiss guidelines [4], including handouts on nutritional education and physical activity [16], and was equipped with a smartphone with the PathMate2 (PM) app [17]. The PM app was developed with the MobileCoach open-source software for health interventions [18, 19], aiming to support behavioural changes in accordance with state-of-the-art multi-component interventions. The effects of earlier versions of the PM app have been evaluated in longitudinal studies [20, 21]. The gamified PM app included two chat channels (Fig. 1): in the first channel, a conversational agent (virtual coach: Anna or Lukas) chatted with patients; in the other channel, HCP (human coaches) and patients were able to chat with each other [17]. The conversational agent chatted daily with the PM participants, encouraging them to achieve challenges like a number of steps per day, performing relaxing breathing exercises [for more details [22]], taking photos of their meals, or answering quality of life questions in order to earn virtual rewards. Most of the time and to allow efficient and reproducible conversational turns, patients were able to respond to Anna/Lukas with predefined answer options. In the app dashboard, the patients could see their progress during the game. Furthermore, a dashboard overview alerted HCP, in case of lack of chat interaction during more than 2 days. Usage time of all mobile apps, and self-reported comfort with and carrying of the smartphone on the body were also recorded.

On the other hand, during this intensive intervention phase, the CON group had an individual multi-component BCI following Swiss guidelines [4] without the PM app. This BCI uses behavioural change techniques such as goal setting, self-monitoring, stimulus control and behavioural contracting to support a healthy lifestyle. The CON received homework exercises based on worksheets. They had to bring their expanding handbook at each hospital visit. During on-site visits, topics like physical activity, diet, mental health and well-being, motivation for healthy choices in everyday life as well as family eating activities and communication habits were discussed.

During the intensive intervention phase of 5.5 months, the PM group had daily interactions with the conversational agent, four on-site visits and two remote counselling sessions via telephone as well as a maximum of 10 min chat time per participant with the human coach, resulting in a total contact time with HCP of approximately 6 h per participant. During the same period the CON group had seven on-site visits with total contact time with HCP of approximately 8 h per participant.

At the end of the intensive intervention phase the use of PM app was ended and the PM group returned the smartphone with the PM App to the HCP.

### Maintenance phase

The maintenance phase served as the self-responsible implementation of a healthy lifestyle according to what had been learned in the intensive intervention phase. It started after the end of the intensive phase and lasted 6 months, including two BCI reflecting sessions, identical in both groups at nine and 12 months after the start of the study. In the 9-month-visit, both groups had a physical examination, a biofeedback session and a BCI dialogue on goal setting and emotions. The final 12-month-visit consisted of a physical examination, a biofeedback session with completion of stress questionnaires and a discussion about maintaining a sustainable healthy lifestyle.

### Biofeedback

All participants performed biofeedback training exercises during hospital visits, under the guidance of HCP, at baseline, and three (data not shown), 5.5 and 12 months after the start of the intervention. Adolescents did a two-minute relaxation exercise: they had to perform abdominal breathing while trying to decrease the amplitude of the skin conductance response, which they could observe in real-time on the computer screen. Skin conductance finger sensors measured the sweat gland activity, which is a sensitive indicator of arousal [23] (NeXus-10 Mark II, Mind Media, Netherlands). Plasma cortisol level, as stress marker, was measured before and after each biofeedback relaxation exercise.

### Outcome measures

Outcomes were assessed at baseline (on average 5 weeks before the start of the Intervention), at the end of the 5.5-month intensive intervention phase (T1), and after the six-month maintenance phase (T2, at 12 months). All measurements were carried out in a non-fasting state and at the same time of day for each subject.

The primary outcome was BMI-SDS. BMI is calculated as weight in kilograms divided by height in meters squared. Since BMI normally increases with age in children and adolescents and taking into account the age- and sex-related skewness of BMI distribution, BMI was adjusted according to the LMS method for sex, age as well as the skewness of BMI distribution, and indicated as standard deviation score (BMI-SDS), identical to BMI z-score, as referred to on the WHO web site (https://www.who.int/tools/growth-reference-data-for-5to19-years/indicators/bmi-for-age) and in Braegger et al. [14]).

Secondary outcomes included body composition, waist-to-height ratio, physical capacities, blood pressure, heart rate and stress assessment. Muscle and fat mass were evaluated by bioelectrical impedance analysis (InBody720) [24, 25]. Waist circumference (WC) was measured with a flexible non-elastic band, and defined as the smallest abdominal circumference between the lowest rib and the upper anterior iliac spine [26]. The intra- and inter-evaluator coefficient of variation of waist circumference measurement in our HCP team was 1.3 and 6.6% respectively (means ±SD: 83.3 ± 1.1 cm and 78.9 ± 5.2 cm, respectively). The measurements of each patient were always performed by the same HCP. Waist-to-height ratio (WHtR), an indicator of obesity-related cardiovascular risk, was calculated using the formula: waist circumference in centimetres divided by body height in centimetres [27]. Puberty was estimated by Tanner stages [28, 29]. Blood pressure (at the right upper arm with an appropriate cuff) and heart rate were measured sitting quietly for 5 min, following standardized procedures [30]. Physical capacities (strength, agility, flexibility, endurance, balance) were assessed by a modified Dordel-Koch-Test (Dordel- Koch-Test [31, 32] plus the plate tapping selected from the Eurofit Test [33]). The modified Dordel-Koch-Test was always conducted by the same physical education teacher for all participants throughout the study and included eight tests:

#### Side jumps

The participant is jumping alternatively right and left over a line as often as possible within 15 s. The test consists of two rounds of 15 s each. The total number of jumps in the two rounds is recorded and the outcome is measured in number of jumps per 30 s.

#### Jump distance

The participant is jumping with both legs as far as possible and landing on both feet. The outcome is measured in centimetres.

#### Sit and reach

With legs fully stretched against a standardized box the participant is reaching with both hands along the top of the box as far as possible. The outcome is measured in centimetres.

#### Balance test

The participant is standing barefoot on a rope on the floor on one leg for 60 s. The outcome is measured in failures, that is when the free leg touches the ground, per 60 s.

#### Sit-ups

The outcome is measured in number of sit-ups per 40 s.

#### Push-ups

The outcome is measured in number of push-ups per 40 s.

#### Six-minute-run

The participant is running for 6 min as far as possible. The outcome is measured in meters.

#### Tap test

The participant is trying to touch 2 disks on a table with the preferred hand alternately as quickly as possible. The outcome is measured in seconds per 25 cycles.

Self-awareness of chronic psychosocial stress was assessed by the Trierer Stress Inventar questionnaire (TICS, T-values, range 15–99, the higher the T-value, the higher the chronic stress level of the patient) [34]. The physical reaction to stress and relaxation was measured by plasma cortisol levels (Chemiluminescence immunoassay, UniCel Dxl 800, Beckman Coulter, in compliance with standard laboratory quality criteria; norm for morning cortisol 140–550 nmol/L, for a cortisol concentration of 166 nmol/L the inter-assay and intra-assay coefficient of variation is 6.7 and 7.9%, respectively), before and after the biofeedback relaxation breathing exercise. The % change of cortisol after relaxation compared to cortisol before relaxation was calculated as 100*(value after – value before) / (value before). Blood samples for the cortisol measurement were scheduled at the same time of day for each subject.

The mental health total score was completed by parents (Strengths and Difficulties Questionnaire, SDQ [35]); it was considered as normal below 13, borderline between 14 and 16 and abnormal above 17 points. As in a national study [6], good school performance predicted a significantly greater decrease in BMI z-score, only this question was selected from KIDSCREEN-52 health-related quality of life questionnaire (HRQoL, “Last week - have you got on well at school?”, Likert scale from 1 “not at all” to 5 “extremely (good)”) [36].

The App usage was evaluated by the number of conversational turns with the conversational agent required to accept a challenge. Adherence was measured as the number of challenges successfully completed divided by the total number of challenges. More detailed data on app usage and satisfaction with the conversational agent has been published in part in other work [22].

### Statistical analyses

Power and sample size were calculated on the basis of the changes observed in our childhood obesity management centre [7], which showed a mean BMI-SDS change (± SD) of − 0.33 ± 0.44 after 1 year of individual multi-component BCI. We expected to achieve similar results with our two study groups. Data simulation from the above distribution showed that 17 patients per group would provide 80% power to detect a significant BMI-SDS change in one of the treatment groups using Wilcoxon signed rank tests. To account for a few dropouts, we aimed to recruit 20 patients per group.

Statistical analyses were performed using rank tests because some outcomes were not normally distributed. The distribution of outcomes across all patients and within each of the treatment groups was described by medians with ranges. *P*-values for the significance of changes from baseline to T1 (D1) or T2 (D2) were calculated using Wilcoxon signed-rank tests. *P*-values for differences between the two groups (at individual time points and for change from baseline to T1 or T2) were obtained with Wilcoxon rank sum tests. Associations between numeric variables were described with Spearman rank correlations. Confidence intervals for these coefficients were derived from the normal approximation obtained with Fisher’s transformation. Associations between categorical variables were described by frequencies and tested with Fisher’s exact tests. A significance level of 0.05 was applied for all tests without correction for multiple testing given the exploratory nature of the analyses performed for the secondary outcomes. Data analysis was performed using the statistical software R, version 3.5.1 (2018-07-02). For the primary outcome we also conducted a last observation carried forward (LOCF) analysis and a responder analysis with Fisher’s exact test (responders defined as participants showing BMI-SDS change < 0). Additional analyses of group differences with generalized estimating equations (GEE) as well as calculations of intra- and intergroup effect sizes were performed for the primary outcome and for physical capacities.

## Results

### Patient characteristics at baseline

**Fig. 2:**
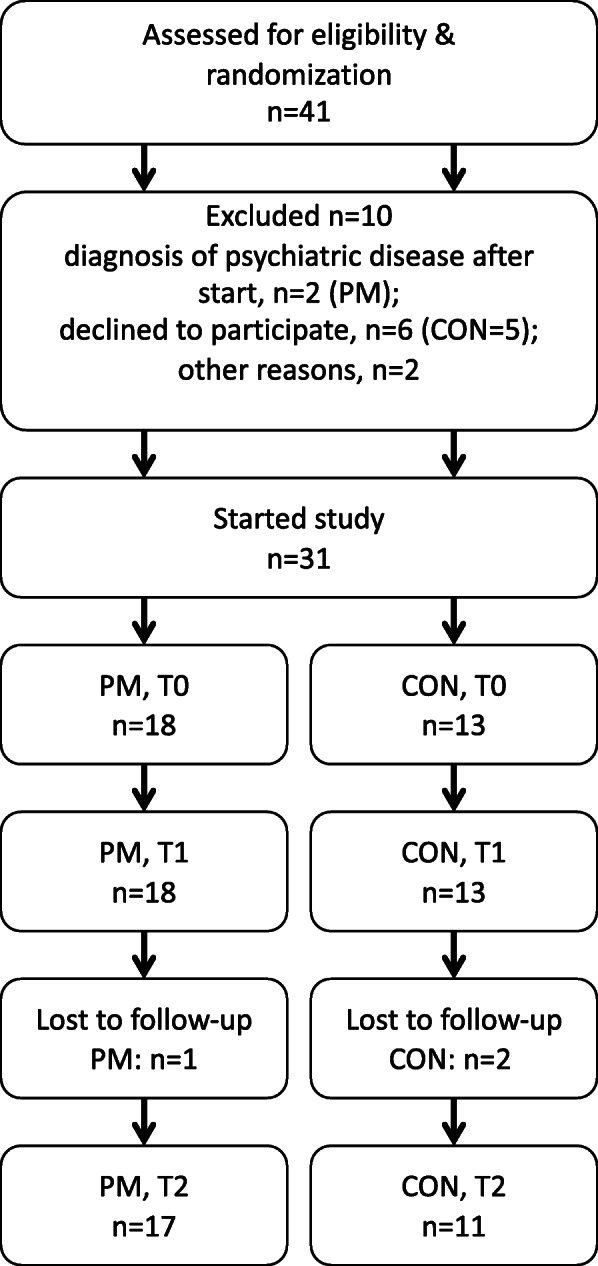
Participants flow throughout the study. Legend Fig. 2: Refer to patient characteristics for further information. PM: PathMate group, CON: Control group, T0: intervention start, T1 and T2: 5.5 and 12 months after intervention start, respectively, n: number of patients

Figure 2 provides a flowchart for enrolment, randomization, intervention and follow-up (maintenance phase) of study participants. Tables 1 and 2 display baseline characteristics. 87% of the participants (27 out of 31) were referred to us from their physician for obesity therapy and 13% contacted us directly wanting to participate in our study. After randomisation and the start of intervention, two patients dropped out due to intercurrent diagnosis of psychiatric disease and thus were excluded from all analyses. Three further patients, who dropped out after the first 5 months of the study, were included in the analyses of outcomes at T1 but excluded in the analysis of outcomes at T2. For all variables except BMI-SDS, the number of patients in the analysis was further reduced by non-compliance with the study plan or physical incapacity due to illness.

**Table 1:** Baseline characteristics before the start of intervention

	Whole Cohort	PM	CON	*p*-value
Age (years)	13.6 (10.9–16.9)	12.6 (11.4–16.9)	13.7 (10.9–16.8)	0.68
BMI (kg/m^2^)	28.8 (22.6–35.7)	28.8 (22.6–35.7)	28.8 (23.5–34.5)	0.83
BMI-SDS	2.5 (1.5–3.5)	2.5 (1.5–3.5)	2.5 (1.5–3.2)	0.74
SDQ total score	12.0 (1.0–25.0)	8.0 (2.0–25.0)	12.6 (1.0–25.0)	0.88
BMI mother (kg/m^2^)	27.3 (20.4–36.2)	27.4 (20.4–36.2)	27.3 (22.3–34.4)	0.98
BMI father (kg/m^2^)	28.4 (21.7–38.0)	28.4 (22.3–38.0)	27.7 (21.7–37.5)	0.67

Results are shown as medians with range. *P*-values for differences between PathMate2 (*PM*) and control (*CON*) group were obtained using Wilcoxon rank sum test

*BMI* body mass index, *BMI-SDS* body mass index standard deviation score, *SDQ* Strengths and Difficulties Questionnaire

**Table 2:** Categorical baseline characteristics with number and percentage of patients in each category

Factor	levels	Whole Cohort	PM	CON	***p***-value
Sex	female	13 (41.9%)	7 (38.9%)	6 (46.2%)	0.73
	male	18 (58.1%)	11 (61.1%)	7 (53.8%)	
Pubertal stage	5 (B5 / G5)	9 (34.6%)	4 (44.4%)	5 (55.6%)	
	4 (B4 / G4)	10 (38.5%)	5 (50%)	5 (50%)	
	3 (B3 / G3)	5 (19.2%)	5 (100%)	0 (0%)	
	1&2 (B1&2 / G1&2)	2 (7.7%)	1 (50%)	1 (50%)	
Psychological/Behavioural problems	no	13 (41.9%)	8 (44.4%)	5 (38.5%)	1.00
	yes	18 (58.1%)	10 (55.6%)	8 (61.5%)	
Physiotherapy	no	29 (93.5%)	18 (100%)	11 (84.6%)	0.17
	yes	2 (6.5%)	0 (0%)	2 (15.4%)	
Psychotherapy	no	28 (90.3%)	15 (83.3%)	13 (100%)	0.25
	yes	3 (9.7%)	3 (16.7%)	0 (0%)	
Nutrition counseling	no	24 (77.4%)	13 (72.2%)	11 (84.6%)	0.67
	yes	7 (22.6%)	5 (27.8%)	2 (15.4%)	
School Performance*	1	1 (3.4%)	1 (6.2%)	0 (0%)	0.81
	2	6 (20.7%)	4 (25.0%)	2 (15.4%)	
	> 3	22 (75.9%)	11 (68.8%)	11 (84.6%)	

*P*-values for differences between PathMate2 (*PM*) and control group (*CON*) were obtained using Fisher’s exact test. Pubertal stages were assessed according to Tanner staging [28, 29]. *Likert scale from 1 “not at all” to 5 “extremely (good)”

The increase of BMI-SDS (median 2.61, range 1.7–3.5) from baseline to the start of the intervention was not statistically significant (*p*-value 0.09). Subjects’ characteristics were similar among groups (Tables 1 and 2), though mental health problems assessed by SDQ total score displayed different distributions. 51.6% of mothers and 45.2% of fathers were of Swiss origin.

Physical performance and capacities were similar in both groups, except CON boys achieving better results in jump distance and 6-min-run compared to PM boys (Additional File 1). With few exceptions, subjects had lower values than the age- and gender-specific references [31, 37].

### Primary and secondary outcomes

**Fig. 3:**
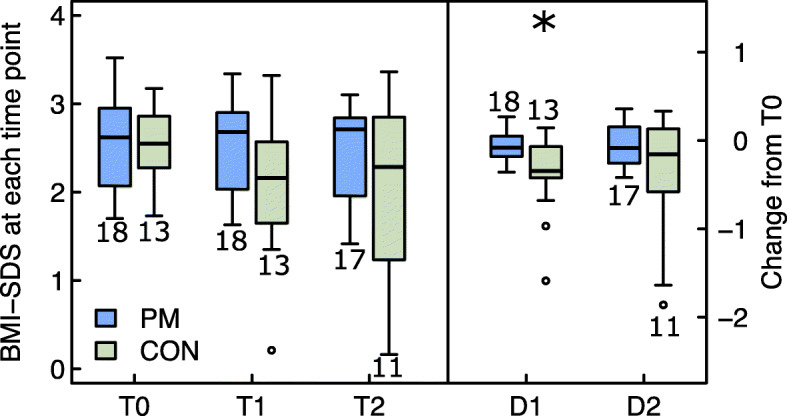
Group comparison of BMI-SDS at the start of intervention (T0), after 5.5 (T1) and 12 months (T2), and changes from T0 to T1 (= D1) and from T0 to T2 (= D2). Legend Fig. 3: ^*^ *p*-values < 0.05 for changes within groups. BMI-SDS: body mass index standard deviation score, PM: PathMate2 group, CON: Control group. The numbers above or below the boxplots indicate the number of patients investigated at each point of time

**Fig. 4:**
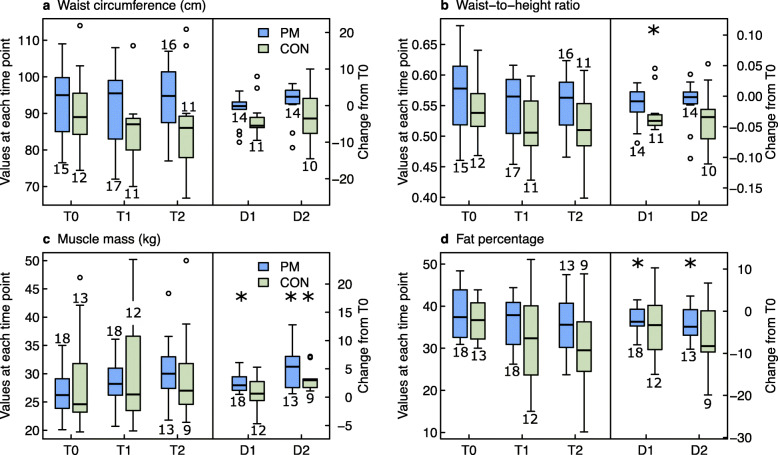
Group comparison of body measurements at the start of intervention (T0), after 5.5 (T1) and 12 months (T2), and changes from T0 to T1 (= D1) and from T0 to T2 (= D2). Legend Fig. 4: ^*^ *p*-values < 0.05 for changes within groups. PM: PathMate2 group, CON: Control group. The numbers above or below the boxplots indicate the number of patients investigated at each point of time

**Fig. 5:**
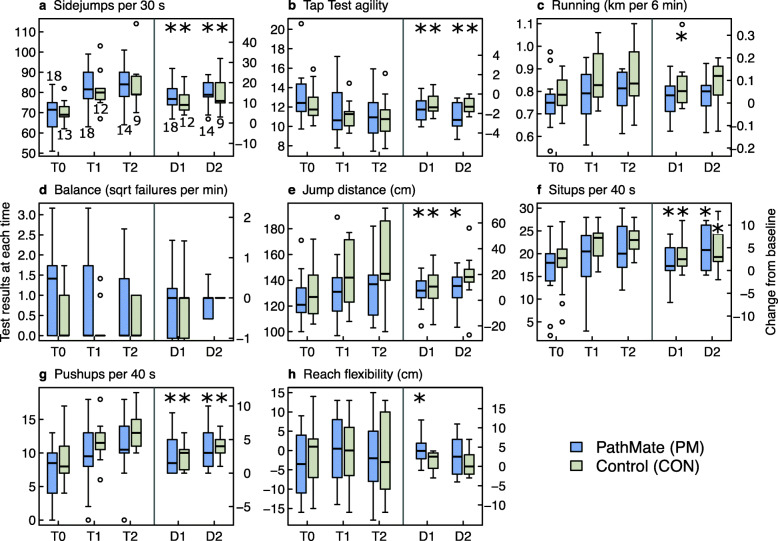
Group comparison of fitness test at the start of intervention (T0), after 5.5 (T1) and 12 months (T2), and changes from T0 to T1 (= D1) and from T0 to T2 (= D2). Legend Fig. 5: ^*^ *p*-values < 0.05 for changes within groups. PM: PathMate2 group, CON: Control group. The numbers above or below the boxplots in Fig. 5a indicate the number of patients investigated at each point of time and can be applied to all fitness tests

**Fig. 6:**
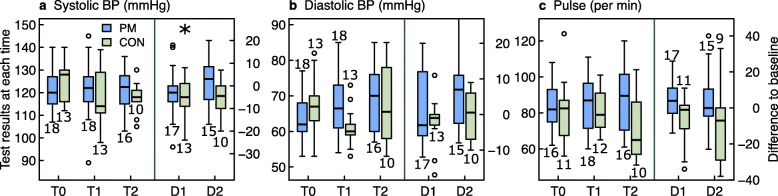
Group comparison of blood pressure (BP) and pulse at the start of intervention (T0), after 5.5 (T1) and 12 months (T2), and changes from T0 to T1 (= D1) and from T0 to T2 (= D2). Legend Fig. 6: ^*^ *p*-values < 0.05 for changes within groups. PM: PathMate2 group, CON: Control group. The numbers above or below the boxplots indicate the number of patients investigated at each point of time

**Fig. 7:**
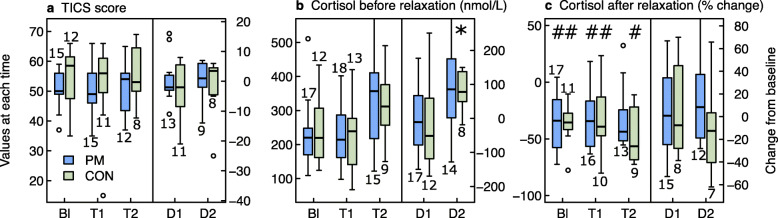
Group comparison of stress parameters at baseline (Bl), after 5.5 (T1) and 12 months (T2), and changes from Bl to T1 (= D1) and from Bl to T2 (= D2). Legend Fig. 7: ^*^ *p*-values < 0.05 for changes within groups. ^#^ p-values < 0.05 for % change after relaxation within groups (whether % change after relaxation differs from 0). Baseline: on average 5 weeks before the start of intervention. Cortisol before relaxation (nmol/L): plasma cortisol before biofeedback relaxation exercise. Cortisol after relaxation (% change): percentage change of plasma cortisol after biofeedback relaxation exercise compared to cortisol before relaxation. BI: baseline, PM: PathMate2 group, CON: Control group, TICS: Trierer Stress Inventar questionnaire. The numbers above or below the boxplots indicate the number of patients investigated at each point of time

Results of the primary and secondary outcomes are presented in Figs. 3, 4, 5, 6, 7 as well as in additional files 2 to 6 and 11.

#### Obesity measures (Figs. 3 & 4, Additional files 2, 3 & 11)

BMI-SDS decreased (responders defined as participants showing BMI-SDS change < 0) in 92% of CON and 61% of the PM group between T0 and T1 (OR = 0.14 [95% CI 0.003 to 1.37]), *p* = 0.095); from T0 to T2 a decrease in BMI-SDS was observed in 64% of CON and in 59% of the PM group (OR = 0.82 [95% CI 0.12 to 4.94] *p* = 1.00), without significant group differences.

In the PM group the median decrease in BMI-SDS from T0 to T1 and from T0 to T2 was not significant (− 0.08, range − 0.4 to + 0.3, *p* = 0.15 and − 0.09, range − 0.4 to + 0.4, *p* = 0.33, respectively, Fig. 3, additional file 2). Controls reduced significantly their BMI-SDS from T0 to T1 (median − 0.35, range − 1.6 to + 0.1, *p* = 0.002), but the BMI-SDS change from T0 to T2 was not significant (median − 0.16, range − 1.9 to + 0.3, *p* > 0.05). Differences between groups were only significant at T1 (*p* = 0.02). The results of the additional analysis with GEE and intra- and intergroup effect sizes for BMI-SDS were consistent with the above rank test results (see Additional file 11).

*Waist-to-height ratio (WHtR)* in the CON group showed a significant decrease from T0 to T1 (0.54, range 0.47 to 0.64 and 0.51, range 0.43 to 0.60, respectively, *p* = 0.04, assessed in only 11 CON subjects; Fig. 4, additional file 3). No other significant changes in WC or WHtR within or between groups were observed.

#### Body composition (Fig. 4, Additional file 3)

The PM group significantly increased its muscle mass and decreased its fat mass at T1 and T2; however, the body fat percentage still remained above the normal range (up to 24% in boys and 35% in girls [25]). The CON showed a significant muscle mass increase after 12 months.

#### Physical capacities (Fig. 5, Additional files 4 & 11)

From T0 to T1 both groups improved significantly their agility (side jumps, tap test) and strength (jump distance, sit-ups, push-ups), without significant differences between groups. At T2, significant further improvements were observed for side jumps, tap test, sit-ups and push-ups in both groups, without significant group difference. The results of the additional analysis with GEE and intra- and intergroup effect sizes for physical capacities were consistent with the above rank test results (see Additional file 11).

#### Cardiovascular parameters (Fig. 6, Additional file 5)

Systolic blood pressure and heart rate tended to be in the upper normal range in the whole cohort at start and more than 50% of the measures were above the upper normal range, without any improvements at T2. Diastolic blood pressure was normal in nearly all adolescents and remained unchanged throughout the study.

#### Stress parameters (Fig. 7, Additional file 6)

Chronic stress (TICS) and plasma cortisol levels were in the middle of normal ranges at baseline investigations. TICS and cortisol levels (before biofeedback) did not differ between groups and there was no significant long-term change during the 1 year of study, with the exception of an increase of cortisol at T2 in 8 CON subjects. However, there was a significant % decrease in cortisol levels after the biofeedback, compared to cortisol before biofeedback, during the study, with the exception of the PM group at T2.

### Adherence to therapy and adverse events

After half a year, 51% of the PM participants were still using the App and 54% were active on achieving challenges. The average app usage rate was 71.5% and the average adherence rate was 57.2% during the first 5.5 months of the study. During this period, all CON kept their hospital appointments, 95% brought, as asked, their handbook to each visit and 68% did the assigned homework.

According to self-reports 3 months after study start, PM subjects carried, on weekdays, 10 h the smartphone with them (median, range 0–21.5), whereas the time the smartphone stayed outside their bedroom at night was 8 h (range 0–13); these results were similar for weekends and did not change during the course of the 5.5 month-app intervention. The average time per day that adolescents spent with their smartphone was 16.2 min, with YouTube and the PM app being the two most frequently used apps (for details, see Additional file 7, Tables a&b). Moreover, the PM app was used, on average, for 48 s per day, which reflects approximately 16% of the overall smartphone usage during the intervention period. Thus, no serious adverse events with respect to smartphone addiction were evident.

### Prediction of therapy success / associations

Therapy success (reduction in BMI-SDS) was associated with neither baseline age, sex, BMI-SDS, psychological or school problems of the participants, nor BMI, foreign nationality or psychological problems of the parents.

From T0 to T1, patients with a greater reduction in BMI-SDS also had a greater reduction in waist circumference and in fat percentage, with little or no increase in muscle mass (analysis performed for the whole cohort, Additional file 8, see most right column in scatter plots). However, changes in BMI-SDS, waist circumference, muscle and fat mass from T0 to T1 were not significantly associated with changes in physical capacities, or with the levels achieved at T0 or T1 (Additional file 8, see first three columns from left to right). Thereby, even if most patients improved their physical fitness and capacities, and many also reduced their BMI-SDS and body fat, the magnitude of these changes varied individually, and these individual variations were not related to each other.

We found a weak positive correlation between changes in chronic stress and changes in waist circumference and BMI-SDS after 12 months (Additional file 9). Otherwise, no significant associations were found between BMI-SDS and stress parameters (TICS scores, cortisol before biofeedback, cortisol change after biofeedback) or changes in BMI-SDS, WC, WtHR and stress changes during the study (data not shown).

## Discussion

The PathMate2 study is one of the first randomized controlled trials assessing the short and long-term effects of a novel mobile application, which aims to encourage healthy lifestyle choices in adolescents with overweight or obesity.

The PathMate2 gamified intervention did not result in a sustained significant change of BMI-SDS. However, it achieved significant and lasting improvements of body fat and physical capacities during the whole 12 months of the study and a high app user engagement. The CON group, having a standardized BCI with more on-site visits, reduced significantly their BMI-SDS and waist-to-height ratio after 5.5 months. Nevertheless, these changes were not sustained up to 12 months after study start. Controls also improved their physical capacities in the long term, without significant group differences.

### Attrition rates, adherence to therapy and adverse events

Attrition rates observed in childhood obesity management programs, mainly in the USA, are reported to reach 27 to 73% [38], while the dropout rates of multi-professional BCI in group setting for children and adolescents in Switzerland range between 10 and 20% [6]. Despite an extensive preliminary assessment and a signed informed consent, but in accordance with the above numbers, 24% of our subjects withdraw between the recruitment and the start of the study. However, 94% of the eligible PM patients who started the study also completed it. Moreover, half of the PM participants were still using the App daily after half a year, in contrast to the high dropout rates reported in digital health interventions with adherence dropping after a few weeks [39, 40]. The high adherence rate seen in the PM group may be explained by the daily encouragement through a peer-appealing conversational agent with real-time feedback, the rewarding game system, the competition with peers and the low threshold of communication with HCP. During the first 5.5 months of the study the CON group also showed a high compliance with 100% attendance at hospital appointments and 85% of the eligible CON patients completing the study. This high adherence to therapy could be attributed to the structured BCI and to the personal counseling from childhood obesity specialists.

The total smartphone app usage time (including PM app), which was 16.2 min per day during the 5.5-month app intervention, remains (even with max. value of 88.6 min per day) clearly below the 2 h and 42 min reported among 13- to 18-year-olds in the U.S. [41]. Additionally, as the most frequently used app was YouTube, we can argue that our app did not promote mobile gaming addictions.

### Primary and secondary outcomes

According to international data [42] only 2% of adolescents exhibit healthy lifestyle behaviours; hence, in pediatric obesity treatment, learning to adopt a sustainable healthy lifestyle is a key goal. In this sense, our study investigated whether a novel smartphone application can support adolescents in adopting long-lasting healthier habits while avoiding yo-yo effects [43]. Although body mass index can only indirectly indicate a healthy behaviour, we have chosen it, in form of BMI-SDS corrected for sex and age, as our primary outcome, as it is the most widely used parameter of measuring obesity.

In the whole cohort, we observed a small but significant improvement in BMI-SDS (− 0.09) after 5.5 months of intervention, and changes were maintained up to one year (− 0.12). These findings suggest that the individual multi-component BCI was effective, similarly to previous studies in various settings and age groups (BMI-SDS change from − 0.06 to − 0.13SD [3]). Interestingly, the number of contact hours in our study was much lower than the 26 h found to be effective through comprehensive and intensive BCI [44]. As a significant decrease in BMI-SDS and waist-to-height ratio were found in the control group only, we conclude, in line with prior work [44], that an intensive personalized behavioural intervention can result in a greater short-term reduction in weight status than a mobile health app program. However, in our study, the effects on BMI-SDS were not maintained at one-year in controls, which may be explained by the wide variations of the CON results and/or the dropout of some patients showing large changes in BMI-SDS from T0 to T1 (two control patients, who dropped out before T2, had a BMI-SDS decrease of − 0.74 at T1, which was larger than that of the entire control group of − 0.37). Nevertheless, the last observation carried forward analysis did not present different results with regard to BMI-SDS (only applied for the primary outcome, Additional file 10). Another explanation for the non-significant BMI-SDS change after one year could be that only two contact hours with HCP between 5.5 and 12 months after study start (maintenance phase) were probably insufficient in order to maintain behavioural changes.

During the intervention, changes in physical capacities and body composition were similar to previous studies [6, 45]. In both groups, we found similar improvements after 5.5 and 12 months in strength measures and agility. The significant improvement of muscular strength, as seen in our study, is of critical importance in improving overall health of adolescents with obesity as well as their comfort in daily life, thus increasing their autonomy [46]. The 6-min-run-test (aerobic fitness) is generally highly and negatively correlated with BMI-SDS [47]; this may explain, why in our study only controls (*n* = 12) showed an improvement in 6-min-run-test at T1 but not at T2. Although balance in obese children has previously been described as impaired [48], our patients showed optimal balance scores already at baseline. The PM intervention resulted in increased muscle mass at 5.5 months and a reduced percentage of fat mass throughout the study. As no correlations between changes in physical capacities and muscle mass were found in our study, it could be argued that the significant increase in muscle mass in both groups could also be a consequence of a synchronized pubertal growth. However, more than two thirds of the participants had a Tanner stage of 4 or 5, and thus had already passed the pubertal stage, when a maximum increase in muscle mass can be expected [49, 50].

In prior Swiss research, and in consistence with our results, systolic hypertension was present in 47.6% of children with obesity [51]. In our study, we did not observe any change in 6-min run test at 12 months, which may explain the absence of change in blood pressure or pulse rate [52].

The effectiveness of IT-mediated obesity interventions in improving health status in this age group remains uncertain, probably due to insufficient intervention duration, small sample size and short-term follow-up. In the 2018 meta-analysis of Ho et al. [53], six RCTs of internet-based self-monitoring interventions revealed a small reduction of BMI or BMI z-score; however, the quality of evidence was low due to the risk of bias and imprecision. On the other hand, in the review of Chen and Wilkosz [54] ten out of 14 studies evaluating the effectiveness of technology-based interventions for obese adolescents showed no effects on BMI or BMI z-score.

Only a few IT-supported interventions have, in line with our results, reported significant positive effects on the percentage of body fat [55, 56], skeletal muscle mass [55], as well as physical activity [57, 58] and muscular fitness [59].

In consideration of the study results, when access to a specialized childhood obesity management center is limited and the adolescent’s primary goal is the improvement of physical status and body composition, PathMate2 app could be a feasible option, if combined with individual counselling sessions. The PathMate2 app could also potentially be implemented in a follow-up program after an intensive obesity intervention. However, if the main focus of an obesity intervention is the reduction of BMI-SDS, the PathMate2 app intervention in its current form would probably be inadequate; in this case more contact hours with HCP and longer or repeated intermittent periods of app usage (longer than 6 months) would probably be needed in order to achieve a satisfactory BMI-SDS reduction.

### Biofeedback and changes of stress parameters

With regard to acute stress response, subjects in our study exhibited significantly lower cortisol levels after the biofeedback relaxation exercises. These findings support the conclusions of prior research [12, 13], that psychophysiological interventions could have a clinical utility in obesity therapy in lowering emotional stress connected with eating disorders.

The present study is, to our knowledge, the only one that has investigated the long-term effect of biofeedback training on obesity related stress response in adolescents. Our hypothesis that the adolescents with obesity who would be most successful in reducing the maladaptive response to chronic stress would also exhibit a decrease in BMI-SDS was not confirmed; however, we found a weak positive correlation between changes in chronic stress (TICS) and changes in waist circumference and BMI-SDS after 12 months. Similarly, the RCT of Emmanouil et al. [60] found no changes in BMI-SDS after an eight-week stress management intervention program in Greek children and adolescents with obesity. Interestingly, baseline markers of acute and chronic stress in our patients, namely cortisol levels and TICS scale, were in the normal range and did not indicate a maladaptive stress response, at least not with the methods used. Although biochemical changes of stress parameters as well as a dysregulation of the cortisol metabolism [61–64] can be found in patients with obesity, obesity is not always related to a hyperresponsive HPA axis and high cortisol levels [61], and the large interindividual variation in glucocorticoid sensitivity [65] could explain the normal cortisol levels in our patients with obesity.

Further research in a larger sample is therefore needed to prove the long-term effectiveness of biofeedback exercises on distress and obesity and identify the best candidates for psychophysiological intervention.

### Prediction of therapy success (change in BMI-SDS)

In our study, we did not find any associations between therapy success and baseline characteristics. However, the two-year Swiss childhood obesity study (KIDSSTEP) [6] as well as 2- to 5-year-follow-up German studies [66, 67] in children with obesity after one-year-interventions showed that the decrease in BMI-z-score was the greatest in children before pubertal age and the smallest in adolescents above 14 years. This association could explain why in our participants, who had a median age of 13.6 years, the BMI-SDS decrease was not significant in the 12-month follow-up.

In the present study, changes in physical capacities were not associated with changes in BMI-SDS, waist circumference, muscle mass or fat mass. Likewise, Reichert et al. [68] concluded that literature data offers only limited support for a causal link between physical activity and adiposity in adolescents; in children, however, the population-based longitudinal observation conducted by Metcalf et al. [69] supported a reverse causality where physical inactivity appeared to be the result of fatness rather than its cause.

### Strengths of the study

The greatest strength of the present study is its randomized controlled design.

Conducting this low-intensity intervention (< 25 contact hours [44]) in a real-world setting with the well-known obesity pitfalls [6] and with adolescents with obesity suffering from challenging somatic and mental comorbidities, makes the results of this study more applicable in clinical practice. The attrition rate after the start of intervention was low and the adherence was high in both groups.

It should be pointed out that data privacy during the patients’ interaction with the PM app was guaranteed using a MobileCoach server located in Switzerland and compliant with Swiss data protection regulations [17, 18].

### Limitations of the study

A limitation of this study is the small sample size due to the well-known recruitment difficulties related to this age group [6, 70, 71] and potential obesity comorbidities (for example psychiatric diseases), the limited time and resources of the families, as shown in the swiss KIDSSTEP study [6], and probably the lack of motivation in participating in a scientific study since the expenses of multi-component BCI in Switzerland are covered by health insurances. Therefore, and because adolescence is characterised by an increased reward-seeking behaviour [72], we decided to reward the best five participants of each group. A potential selection bias due to the attractivity of the smartphone could explain the drop out of five patients after being randomized in the control group and signing the consent forms (Fig. 2, hypothesis: the “less motivated” CON dropped out while the “more motivated” CON chose to continue the study despite not getting into “the smartphone group”). The selection of motivated adolescents might be associated with a better compliance of the control group. Lastly, although co-operating psychologists recommend the use of Trierer Stress Inventar questionnaire to evaluate stress in adolescents from the age of 13 years, TICS is validated on a population above 16 years of age. Otherwise, all other outcome measures were based on objective and validated methods for the age group investigated.

## Conclusions and future research directions

In conclusion, our study results support the effectiveness of the PathMate2 app, combined with standardized counselling, in improving physical capacities, lowering body fat percentage and increasing muscle mass in adolescents with overweight or obesity. These significant and lasting improvements were similar to those achieved in the control group receiving a standardized multi-component behaviour changing intervention with more on-site visits. Thus, when the focus is on improving physical status and body composition but access to a specialized childhood obesity management center is limited, the PathMate2 app may offer a novel healthcare solution, if combined with individual counselling session in a primary care setting. Although, both the PathMate 2 group and controls exhibited a reduction of BMI-SDS during the study, the controls showed a temporary significant BMI-SDS reduction after 5.5 months. Therefore, we assume, that when the intervention focus is on weight reduction, then an intensive BCI with more contact hours with HCP and longer or intermittently repeated periods of app usage is probably more appropriate.

Though chronic stress was not found to be a major determinant of obesity in adolescents of the present study, biofeedback relaxation exercises could be a valuable complementary tool for adolescents with obesity and eating disorders or emotional eating. However, the long-term effects of biofeedback exercises on stress and obesity in youth remain to be evaluated in a larger cohort.

While on the long term, mobile app interventions may become affordable and cost effective, at this stage, the design and technical as well as health professional support of such systems is highly demanding and expensive. In contrast to their use in prevention programs, digital health interventions for adolescents with overweight or obesity could only be effective, and ethically justified, if combined with other behaviour changing interventions conducted by trained HCP and tailored to the patient’s needs. Therefore, a careful baseline assessment of physical and mental status should be performed in order to propose an individualized obesity treatment. In addition, a careful monitoring should be established to follow therapeutic progress and prevent an excessive use of the smartphone.

Childhood is a unique window of opportunity, when treatment can have a lifetime impact on health and quality of life and prevent long-term disability and reduced work productivity. Based on the PathMate2 application, conversational agent-based telemedicine interventions could be implemented for scalable childhood obesity management or follow-up. This approach is particularly interesting during the current COVID-19 pandemic, as digital approaches are rapidly becoming an essential part of teletherapy [73], overcoming the restrictions imposed by governments, while offering modern health care access for more patients and families.

## Supplementary Information

**Additional file 1.** Table a. Physical performance and capacities (modified Dordel-Koch-Test) at baseline in girls. Table b. Physical performance and capacities (modified Dordel-Koch-Test) at baseline in boys

**Additional file 2.** Table – Median BMI-SDS values at each point time and change to intervention start (D1, D2).

**Additional file 3.** Table – Median values of body composition at each point time and change to intervention start (D1, D2).

**Additional file 4.** Table – Median values of fitness tests at each point time and change to intervention start (D1, D2).

**Additional file 5.** Table – Median values of blood pressure and pulse at each point time and change to intervention start (D1, D2).

**Additional file 6.** Table – Median values of TICS and cortisol at each time point and change to baseline (D1, D2).

**Additional file 7. **App usage analytics – Table a. Smartphone app and PathMate2 app usage during the intervention period, i.e. 162 days (*N* = 11). Table b. Most-frequently used smartphone apps during the intervention period, i.e. 162 days (*N* = 11).

**Additional file 8.** Figure – Associations between overall standardized fitness level and changes in BMI-SDS and body composition measures from T0 to T1.

**Additional file 9.** Figure – Associations between changes in TICS and changes in BMI-SDS and waist circumference from T0 to T2.

**Additional file 10. **Table – Median values for BMI-SDS with last observation carried forward analysis at each time point, improvement to baseline (D1, D2) and *p*-values for changes across patients of each group and for group difference.

**Additional file 11.** Table – Analyses of group differences with generalized estimating equations (GEE) and calculations of intra- and intergroup effect sizes for the primary outcome and for physical capacities.

## Data Availability

Expect for a part of the association data (when no association between outcomes was found), all other data generated or analysed during this study are included in this published article and its supplementary information files. Association data not shown can be available from the corresponding author on reasonable request.

## Abbreviations

Bl: Baseline
BCI: Behaviour-changing intervention
BMI: Body mass index
BMI-SDS or BMI z-score: Body mass index standard deviation score
BP: Blood pressure
CI: Confidence interval
CON: Control group
GEE: Generalized estimating equations
HCP: Health care professionals
HIS: Health information systems
HRQoL: Health-related quality of life questionnaire
IT: Information technology
OD: Odds ratio
PathMate2 app: PM app
PM: PathMate2 group
RCT: Randomized controlled study
SDQ: Strengths and difficulties questionnaire
TICS: Trierer stress inventar questionnaire
WC: Waist circumference
WHtR: Waist-to-height ratio

## References

[1] Brown T, Moore THM, Hooper L, Gao Y, Zayegh A, Ijaz S et al. Interventions for preventing obesity in children (Review). Cochrane Database Syst Rev. 2019; Issue 7. Art. No.: CD001871; doi:10.1002/14651858.CD001871.pub4. 10.1002/14651858.CD001871.pub4 PMC6646867 31332776

[2] Reilly JJ, Kelly J (2011). Long-term impact of overweight and obesity in childhood and adolescence on morbidity and premature mortality in adulthood: systematic review. Int J Obes.

[3] Ells LJ, Rees K, Brown T, Mead E, Al-Khudairy L, Azevedo L. Interventions for treating children and adolescents with overweight and obesity: an overview of Cochrane reviews. Int J Obes (Lond). 2018;42(11):1823–1833; doi: 10.1038/s41366-018-0230-y. Epub 2018 Oct 9. Erratum in: Int J Obes (Lond). 2019 Apr 2. 10.1038/s41366-018-0230-y 30301964

[4] Sempach R, Farpour-Lambert NJ, L’Allemand D, Laimbacher J (2007). Therapie des adipösen Kindes und Jugendlichen: Vorschläge für multi­professionelle Therapieprogramme. (Therapy of the obese child and adolescent: Suggestions for multi-professional therapy programs. Article in German.). Paediatrica.

[5] Farpour-Lambert NJ, Martin XE, Bucher Della Torre S, von Haller L, Ells LJ, Herrmann FR (2019). Effectiveness of individual and group programmes to treat obesity and reduce cardiovascular disease risk factors in pre-pubertal children. Clin Obes.

[6] L’Allemand D, Kirchhoff E, Bolten M, Zumbrunn A, Sempach R, Farpour-Lambert N (2012). Evaluation of the treatment of overweight children and adolescents in Switzerland: KIDSSTEP interim analysis of multi-professional group therapy programs until may 1st, 2012. Paediatrica..

[7] Maron L, Maeder M, Kirchhoff E, Ardelt-Gattinger E, l’Allemand D, Laimbacher J. Individual therapy equals group therapy in significantly improving mental and physical health in obese children. Swiss Med Wkly. 2014;144(Suppl 203):20.

[8] Silva BM, Rodrigues JJ, de la Torre DI, López-Coronado M, Saleem K (2015). Mobile-health: a review of current state in 2015. J Biomed Inform.

[9] Wang Y, Xue H, Huang Y, Huang L, Zhang D (2017). A systematic review of application and effectiveness of mHealth interventions for obesity and diabetes treatment and self-management. Adv Nutr.

[10] Torres SJ, Nowson CA (2007). Relationship between stress, eating behavior, and obesity. Nutrition..

[11] Nederkoorn C, Jansen E, Mulkens S, Jansen A (2007). Impulsivity predicts treatment outcome in obese children. Behav Res Ther.

[12] Jordanova NP (2000). Psychological characteristics and biofeedback mitigation in preadolescents with eating disorders. Pediatr Int.

[13] Teufel M, Stephan K, Kowalski A, Käsberger S, Enck P, Zipfel S (2013). Impact of biofeedback on self-efficacy and stress reduction in obesity: a randomized controlled pilot study. Appl Psychophysiol Biofeedback.

[14] Braegger C, Jenni O, Konrad D, Molinari L (2011). New growth charts for Switzerland. Paediatrica.

[15] van Egmond-Froehlich A, Bullinger M, Holl RW, Hoffmeister U, Mann R, Goldapp C (2012). The hyperactivity/inattention subscale of the strengths and difficulties questionnaire predicts short- and long-term weight loss in overweight children and adolescents treated as outpatients. Obes Facts.

[16] Stachow R, Stübing K, von Egmont-Fröhlich A, Vahabzadeh Z, Jaeschke R, Kuhn-Dost A, et al. Leichter, aktiver, gesünder - interdisziplinäres Konzept für die Schulung übergewichtiger oder adipöser Kinder und Jugendlicher. Trainermanual. (Lighter, more active, healthier - interdisciplinary concept for training overweight or obese children and adolescents. Coach manual in German.) Aid infodienst, Bonn, Germany; 2007.

[17] Kowatsch T, Nißen MK, Shih C-H I, Rüegger D, Volland D, Filler A, et al. Text-based Healthcare Chatbots supporting patient and health professional teams: Preliminary results of a randomized controlled trial on childhood obesity. Persuasive Embodied Agents for Behavior Change (PEACH2017) Workshop, co-located with the 17th International Conference on Intelligent Virtual Agents (IVA 2017), Stockholm, Sweden. 2017. 10.3929/ethz-b-000218776.

[18] Kowatsch T, Volland D, Shih C-H I, Rüegger D, Künzler F, Barata F et al. Design and evaluation of a mobile chat app for the open source behavioral health intervention platform MobileCoach. In: Maedche A., Vom Brocke J., Hevner A. (eds). Designing the Digital Transformation. DESRIST 2017. Lecture notes in computer science, vol 10243. Springer, Cham.

[19] Filler A, Kowatsch T, Haug S, Wahle F, Staake T and Fleisch E. MobileCoach: A novel open source platform for the design of evidence-based, scalable and low-cost behavioral health interventions: Overview and preliminary evaluation in the public health context. Wireless Telecommunications Symposium (WTS), New York, NY, 2015, pp. 1–6; doi.org/10.1109/WTS.2015.7117255.

[20] Kowatsch T, Maass W, Pletikosa Cvijikj I, Büchter D, Brogle B, Dintheer A, et al. Design of a health information system enhancing the performance of obesity expert and children teams. 22nd European Conference on Information Systems (ECIS), Tel Aviv, Israel. 2014.

[21] Xu R, Pletikosa Cvijikj I, Kowatsch T, Michahalles F, Büchter D, Brogle B (2014). Tell Me What to Eat – Design and evaluation of a mobile companion helping children and their parents to plan nutrition intake. European Conference of Ambient Intelligence, Eindhoven, The Netherlands, 2014.

[22] Shih C-H I, Tomita N, Lukic YX, Reguera ÁH, Fleisch E, Kowatsch T (2019). Breeze: Smartphone-based acoustic real-time detection of breathing phases for a gamified biofeedback breathing training. Proc ACM Interact Mob Wearable Ubiquitous Technol.

[23] Boucsein W. Electrodermal activity. 2nd edition. Springer; 2012.

[24] Kyle UG, Bosaeus I, De Lorenzo AD, Deurenberg P, Elia M, Gómez JM, et al. Composition of the ESPEN working group. Bioelectrical impedance analysis-part I: review of principles and methods. ESPEN guidelines. Clin Nutr. 2004;23(5):1226–43. 10.1016/j.clnu.2004.06.004. 10.1016/j.clnu.2004.06.004 15380917

[25] Boot AM, Bouquet J, de Ridder MA, Krenning EP, de Muinck Keizer-Schrama SM (1997). Determinants of body composition measured by dual-energy X-ray absorptiometry in Dutch children and adolescents. Am J Clin Nutr.

[26] l’Allemand D, Farpour-Lambert NJ, Laimbacher J (2006). Definition, diagnostisches Vorgehen und Therapie- Indikationen bei Übergewicht im Kindes- und Jugend-alter: Ein Vorschlag für Leitlinien. (definition, diagnostic procedure and therapeutic indications for obesity in children and adolescents: a proposal for guidelines. Article in German). Paediatrica..

[27] Petroff D, Kromeyer-Hauschild K, Wiegand S, l'Allemand-Jander D, Binder G, Schwab KO, et al. (2015). Introducing excess body weight in childhood and adolescence and comparison with body mass index and waist-to-height ratio. Int J Obes.

[28] Marshall WA, Tanner JM (1969). Variations in pattern of pubertal changes in girls. Arch Dis Child.

[29] Marshall WA, Tanner JM (1970). Variations in the pattern of pubertal changes in boys. Arch Dis Child.

[30] National High Blood Pressure Education Program Working Group on High Blood Pressure in Children and Adolescents (2004). The fourth report on the diagnosis, evaluation, and treatment of high blood pressure in children and adolescents. Pediatrics.

[31] Dordel S, Koch B (2004) Basistest zur Erfassung der motorischen Leistungsfähigkeit von Kindern und Jugendlichen. Deutsche Sporthochschule Köln (test for the assessment of motor performance of children and adolescents. German Sport University Cologne, Germany, article in German) https://fitnessolympiade.de/dkt_test?menuIndex=2

[32] Lämmle C, Kobel S, Wartha O, Wirt T, Steinacker JM. Intervention effects of a school-based health promotion program on children’s motor skills. J Public Health. 2016;24:185–92. 10.1007/s10389-016-0715-x. 10.1007/s10389-016-0715-x PMC4882358 27340615

[33] Council of Europe, Committee for the Development of Sport. EUROFIT: Handbook for the EUROFIT Tests of Physical Fitness. Strasbourg, 1983.

[34] Schulz P, Schlotz W, Becker P (2004). Trierer Inventar zum chronischen stress (TICS) [Trier inventory for chronic stress (TICS)].

[35] Goodman R (1999). The extended version of the strengths and difficulties questionnaire as a guide to child psychiatric caseness and consequent burden. J Child Psychol Psychiatry.

[36] Ravens-Sieberer U, Gosch A, Rajmil L, Erhart M, Bruil J, Power M, Duer W, Auquier P, Cloetta B, Czemy L, Mazur J, Czimbalmos A, Tountas Y, Hagquist C, Kilroe J, KIDSCREEN Group (2008). The KIDSCREEN-52 quality of life measure for children and adolescents: psychometric results from a cross-cultural survey in 13 European countries. Value Health.

[37] Schmid M, Romann M, Kriemler S, Zahner L. Wie Kann Die Fitness von Schulkindern Gemessen Werden? Schweizerische Zeitschrift Für «Sportmedizin Und Sporttraumatologie». 2007; 55 (2), 52–61. (How to measure the fitness of school children. Swiss Journal for Sports Medicine and Sports Traumatology, Article in German).

[38] Skelton JA, Beech BM (2011). Attrition in paediatric weight management: a review of the literature and new directions. Obes Rev.

[39] Ghelani DP, Moran LJ, Johnson C, Mousa A, Naderpoor N (2020). Mobile Apps for weight management: A review of the latest evidence to inform practice. Front Endocrinol (Lausanne).

[40] Lin PH, Grambow S, Intille S, Gallis JA, Lazenka T, Bosworth H, Voils CL, Bennett GG, Batch B, Allen J, Corsino L, Tyson C, Svetkey L (2018). The association between engagement and weight loss through personal coaching and cell phone interventions in young adults: randomized controlled trial. JMIR Mhealth Uhealth.

[41] Rideout V (2016). Measuring time spent with media: the common sense census of media use by US 8- to 18-year-olds. J Child Media.

[42] Marques A, Bordado J, Tesler R, Demetriou Y, Sturm DJ, de Matos MG (2020). A composite measure of healthy lifestyle: a study from 38 countries and regions from Europe and North America, from the health behavior in school-aged children survey. Am J Hum Biol.

[43] Dulloo AG, Montani JP (2015). Pathways from dieting to weight regain, to obesity and to the metabolic syndrome: an overview. Obes Rev.

[44] Grossman DC, Bibbins-Domingo K, Curry SJ, Barry MJ, Davidson KW, Doubeni CA, US Preventive Services Task Force (2017). Screening for obesity in children and adolescents: US preventive services task force recommendation statement. JAMA.

[45] Dao HH, Frelut ML, Peres G, Bourgeois P, Navarro J (2004). Effects of a multidisciplinary weight loss intervention on anaerobic and aerobic aptitudes in severely obese adolescents. Int J Obes Relat Metab Disord.

[46] Thivel D, Ring-Dimitriou S, Weghuber D, Frelut ML, O'Malley G (2016). Muscle strength and fitness in pediatric obesity: a systematic review from the European childhood obesity group. Obes Facts.

[47] Calders P, Deforche B, Verschelde S, Bouckaert J, Chevalier F, Bassle E, Tanghe A, de Bode P, Franckx H (2008). Predictors of 6-minute walk test and 12-minute walk/run test in obese children and adolescents. Eur J Pediatr.

[48] Bataweel E (2020). A, and Alaa I Ibrahim. Balance and musculoskeletal flexibility in children with obesity: a cross-sectional study. Ann Saudi Med.

[49] Degache F, Richard R, Edouard P, Oullion R, Calmels P. The relationship between muscle strength and physiological age: a cross-sectional study in boys aged from 11 to 15. Ann Phys Rehabil Med 2010;53(3);180–188; https://doi.org/10.1016/j.rehab.2010.02.001. 10.1016/j.rehab.2010.02.001 20226753

[50] Xu L, Nicholson P, Wang Q, Alén M, Cheng S (2009). Bone and muscle development during puberty in girls: a seven-year longitudinal study. J Bone Miner Res.

[51] Maggio AB, Aggoun Y, Marchand LM, Martin XE, Herrmann F, Farpour-Lambert NJ (2008). Associations among obesity, blood pressure, and left ventricular mass. J Pediatr.

[52] Farpour-Lambert NJ, Aggoun Y, Marchand LM, Martin XE, Herrmann FR, Beghetti M (2009). Physical activity reduces systemic blood pressure and improves early markers of atherosclerosis in pre-pubertal obese children. J Am Coll Cardiol.

[53] Ho TJH, Lee CCS, Wong SN, Lau Y (2018). Internet-based self-monitoring interventions for overweight and obese adolescents: a systematic review and meta-analysis. Int J Med Inform.

[54] Chen JL, Wilkosz ME (2014). Efficacy of technology-based interventions for obesity prevention in adolescents: a systematic review. Adolesc Health Med Ther.

[55] Jun MK, Ha JY (2016). Effect of smartphone apps applying BodyThink program on obesity in adolescent girls. J Korean Acad Nurs.

[56] Mohammed Nawi A, Che Jamaludin FI (2015). Effect of internet-based intervention on obesity among adolescents in Kuala Lumpur: a school-based cluster randomised trial. Malays J Med Sci.

[57] Ruotsalainen H, Kyngäs H, Tammelin T, Heikkinen H, Kääriäinen M (2015). Effectiveness of Facebook-delivered lifestyle counselling and physical activity self-monitoring on physical activity and body mass index in overweight and obese adolescents: a randomized controlled trial. Nurs Res Pract.

[58] Whittemore R, Jeon S, Grey M (2013). An internet obesity prevention program for adolescents. J Adolesc Health.

[59] Smith JJ, Morgan PJ, Plotnikoff RC, Dally KA, Salmon J, Okely AD, Finn TL, Lubans DR (2014). Smart-phone obesity prevention trial for adolescent boys in low-income communities: the ATLAS RCT. Pediatrics..

[60] Emmanouil CC, Pervanidou P, Charmandari E, Darviri C, Chrousos GP (2018). The effectiveness of a health promotion and stress-management intervention program in a sample of obese children and adolescents. Hormones (Athens).

[61] Incollingo Rodriguez AC, Epel ES, White ML, Standen EC, Seckl JR, Tomiyama AJ (2015). Hypothalamic-pituitary-adrenal axis dysregulation and cortisol activity in obesity: a systematic review. Psychoneuroendocrinology..

[62] Wiegand S, Richardt A, Remer T, Wudy SA, Tomlinson JW, Hughes B (2007). Reduced 11-hydroxysteroid dehydrogenase type 1 activity in obese boys. (Reduced 11-hydroxysteroid dehydrogenase type 1 activity in obese boys. Article in German.). Eur J Endocrinol.

[63] Messerli-Bürgy N, Horsch A, Schindler C, Boichat A, Kriemler S, Munsch S, Crottet B, Marquez-Vidal PM, Borghini A, Puder JJ (2019). Influence of acute physical activity on stress reactivity in obese and normal weight children: a randomized controlled trial. Obes Facts..

[64] Noppe G, van den Akker EL, de Rijke YB, Koper JW, Jaddoe VW, van Rossum EF (2016). Long-term glucocorticoid concentrations as a risk factor for childhood obesity and adverse body-fat distribution. Int J Obes.

[65] van der Valk ES, Savas M, van Rossum EFC (2018). Stress and obesity: are there more susceptible individuals?. Curr Obes Rep.

[66] Reinehr T, Kleber M, Lass N, Toschke AM (2010). Body mass index patterns over 5 y in obese children motivated to participate in a 1-y lifestyle intervention: age as a predictor of long-term success. Am J Clin Nutr.

[67] Wiegand S, Keller KM, Lob-Corzilius T, Pott W, Reinehr T, Röbl M, Stachow R, Tuschy S, Weidanz I, Widhalm K, de Zwaan M, Holl RW (2014). Predicting weight loss and maintenance in overweight/obese pediatric patients. Horm Res Paediatr.

[68] Reichert FF, Baptista Menezes AM, Wells JC, Carvalho Dumith S, Hallal PC (2009). Physical activity as a predictor of adolescent body fatness: a systematic review. Sports Med.

[69] Metcalf BS, Hosking J, Jeffery AN, Voss LD, Henley W, Wilkin TJ (2011). Fatness leads to inactivity, but inactivity does not lead to fatness: a longitudinal study in children (EarlyBird 45). Arch Dis Child.

[70] Vidmar AP, Pretlow R, Borzutzky C, Wee CP, Fox DS, Fink C, Mittelman SD (2019). An addiction model-based mobile health weight loss intervention in adolescents with obesity. Pediatr Obes.

[71] Pretlow RA, Stock CM, Allison S, Roeger L (2015). Treatment of child/adolescent obesity using the addiction model: a smartphone app pilot study. Child Obes.

[72] Galvan A (2010). Adolescent development of the reward system. Front Hum Neurosci.

[73] Badawy SM, Radovic A (2020). Digital approaches to remote pediatric health care delivery during the COVID-19 pandemic: existing evidence and a call for further research. JMIR Pediatr Parent.

