# Supplementary material for: Effects of a novel mobile health intervention compared to a multi-component behaviour changing program on body mass index, physical capacities and stress parameters in adolescents with obesity: a randomized controlled trial
Source: BMC Pediatr. 2021 Jul 9;21:308. doi: 10.1186/s12887-021-02781-2 (PMC8266630; doi:10.1186/s12887-021-02781-2)
Supplement: Supplementary file 1 — Additional file 1. Table a. Physical performance and capacities (modified Dordel-Koch-Test) at baseline in girls. Table b. Physical performance and capacities (modified Dordel-Koch-Test) at baseline in boys [file 12887_2021_2781_MOESM1_ESM.pdf]

Additional file 1

Table a. Physical performance and capacities (modified Dordel-Koch-Test) at baseline in girls

**Test**	**PM**	**CON**	**Normal values**
Side jumps [n/30 sec]	72 (62 – 82)	68 (64 – 74)	72
Tap Test [sec/25 cycles] [a]	11.9 ± 1.9 [a]	13 ± 1.6 [a]	17.76 ± 2.94 [a]
6-minute-run [meter]	763.8 (762.5 – 912-5)	763.8 (657.5 – 875)	981
Balance [failures/min]	0.5 (0 – 3)	0.5 (0 – 3)	0
Jump distance [cm]	128.5 (108 – 172)	123.5 (106 – 130)	145
Sit-ups [n/40 sec]	18.5 (18 – 27)	14 (5 – 20)	20
Push-ups [n/40 sec]	9.0 (6 – 17)	8.5 (4 – 11)	8
Sit & reach [cm]	3 (-15 – 6)	2.5 (-11 – 14)	5

Results are shown as medians with range, or mean with SD when indicated [a].

PM: PathMate2 group, CON: Control group.

Normal test values are for 13-13,9 year-old-girls [1] except for tap test (normal values for 11-year-old girls [2]).

Table b. Physical performance and capacities (modified Dordel-Koch-Test) at baseline in boys

Test	PM	CON	Normal values
Side jumps [n/30 sec]	71 (51 – 84)	71 (67 – 74)	79
Tap Test [sec/25 cycles][a]	13.5 ± 2.6 [a]	11.7 ± 2.1 [a]	17.32 ± 2.3 [a]
6-minute-run [meter]	725 (535 – 810)	786.3* (737.5 – 975)	1071
Balance [failures/min]	2 (0 – 10)	0.5 (0 – 4)	0
Jump distance [cm]	117 (100 – 149)	142* (120 – 171)	160
Sit-ups [n/40 sec]	18 (2 – 26)	21.5 (13 – 20)	24
Push-ups [n/40 sec]	7 (0 – 12)	8 (4 – 13)	11
Sit & reach [cm]	-11 (-16 – 9)	-6.5 (-9 – 8)	-4

Results are shown as medians with range, or mean with SD when indicated [a].

PM: PathMate2 group, CON: Control group.

* PM different from CON (p <0.05).

Normal test values are for 13-13,9 year-old-boys [1] except for tap test (normal values for 11-year-old boys [2]).

1. Dordel S, Koch B (2004) Basistest zur Erfassung der motorischen Leistungsfähigkeit von Kindern und Jugendlichen. Deutsche Sporthochschule Köln (Test for the assessment of motor performance of children and adolescents. German Sport University Cologne, Germany, Article in German) https://fitnessolympiade.de/dkt_test?menuIndex=2

2. Schmid M, Romann M, Kriemler S, Zahner L. Wie Kann Die Fitness von Schulkindern Gemessen Werden? Schweizerische Zeitschrift Für «Sportmedizin Und Sporttraumatologie». 2007; 55 (2), 52–61. (How to measure the fitness of school children. Swiss Journal for Sports Medicine and Sports Traumatology, Article in German)
