# Supplementary material for: Effects of a novel mobile health intervention compared to a multi-component behaviour changing program on body mass index, physical capacities and stress parameters in adolescents with obesity: a randomized controlled trial
Source: BMC Pediatr. 2021 Jul 9;21:308. doi: 10.1186/s12887-021-02781-2 (PMC8266630; doi:10.1186/s12887-021-02781-2)
Supplement: Supplementary file 2 — Additional file 2. Table – Median BMI-SDS values at each point time and change to intervention start (D1, D2). [file 12887_2021_2781_MOESM2_ESM.pdf]

Additional file 2 – Median values for BMI-SDS at each point time and change to intervention start (D1, D2).

			**T0**	**T1**	**T2**	**D1 (change T1–T0)**	**D2 (change T2–T0)**
BMI-SDS	PM	median (range)	2.62 (1.7 to 3.5)	2.68 (1.6 to 3.3)	2.71 (1.4 to 3.1)	-0.08 (-0.4 to 0.3)	-0.09 (-0.4 to 0.4)
		n	18	18	17	18	17
	CON	median (range)	2.55 (1.7 to 3.2)	2.16 (0.2 to 3.3)	2.28 (0.2 to 3.4)	-0.35* (-1.6 to 0.1)	-0.16 (-1.9 to 0.3)
		n	13	13	11	13	11
		p-value[a] (group differences)	0.65	0.12	0.48	0.02	0.43

* p-values <0.05

[a] p-values testing the significance of group differences at each time point, and of group differences in change. Null hypothesis: medians of both groups are equal.

BMI-SDS: body mass index standard deviation score, PM: PathMate group, CON: Control group, T0: intervention start, T1 and T2: 5.5 and 12 months after intervention start, respectively
