# Supplementary material for: Effects of a novel mobile health intervention compared to a multi-component behaviour changing program on body mass index, physical capacities and stress parameters in adolescents with obesity: a randomized controlled trial
Source: BMC Pediatr. 2021 Jul 9;21:308. doi: 10.1186/s12887-021-02781-2 (PMC8266630; doi:10.1186/s12887-021-02781-2)
Supplement: Supplementary file 3 — Additional file 3. Table – Median values of body composition at each point time and change to intervention start (D1, D2). [file 12887_2021_2781_MOESM3_ESM.pdf]

Additional file 3 – Median values of body composition at each point time and change to intervention start (D1, D2).

			**T0**	**T1**	**T2**	**D1 (change T1–T0)**	**D2 (change T2–T0)**
**Waist circum-ference (cm)**	PM	median (range)	95.0 (76 to 109)	95.5 (72 to 108)	94.8 (77 to 107)	-0.1 (-10 to 4)	2.5 (-12 to 6)
		n	15	17	16	14	14
	CON	median (range)	89.0 (74 to 114)	87.0 (70 to 108)	86.0 (67 to 113)	-5.5 (-10 to 8)	-3.5 (-14 to 10)
		n	12	11	11	11	10
		p-value[a] (group differences)	0.464	0.115	0.093	0.17	0.079
**Waist-to-height ratio**	PM	median (range)	0.578 (0.46 to 0.68)	0.565 (0.45 to 0.62)	0.563 (0.47 to 0.62)	-0.008 (-0.08 to 0.02)	-0.001 (-0.10 to 0.04)
		n	15	17	16	14	14
	CON	median (range)	0.538 (0.47 to 0.64)	0.506 (0.43 to 0.60)	0.510 (0.40 to 0.61)	-0.040* (-0.05 to 0.05)	-0.034 (-0.11 to 0.05)
		n	12	11	11	11	10
		p-value[a] (group differences)	0.399	0.059	0.121	0.183	0.064
**Muscle mass (kg)**	PM	median (range)	26.2 (20 to 35)	28.2 (21 to 36)	30.0 (22 to 44)	2.1** (0 to 6)	5.4** (1 to 13)
		n	18	18	13	18	13
	CON	median (range)	24.6 (20 to 47)	26.4 (20 to 50)	27.0 (21 to 50)	0.6 (-5 to 5)	3.0** (1 to 7)
		n	13	12	9	12	9
		p-value[a] (group differences)	0.984	0.626	0.242	0.034	0.442

**Fat percentage**	PM	median (range)	37.4 (31 to 48)	37.9 (26 to 44)	35.6 (24 to 48)	-2.5* (-8 to 3)	-3.7* (-9 to 4)
		n	18	18	13	18	13
	CON	median (range)	36.7 (30 to 44)	32.3 (15 to 51)	29.5 (10 to 48)	-3.3 (-15 to 10)	-8.3 (-20 to 7)
		n	13	12	9	12	9
		p-value[a] (group differences)	0.32	0.24	0.23	0.51	0.43

* p-values <0.05, ** p-values <0.001
[a] p-values testing the significance of group differences at each time point, and of group differences in change. Null hypothesis: medians of both groups are equal.
PM: PathMate group, CON: Control group, T0: intervention start, T1 and T2: 5.5 and 12 months after intervention start, respectively
