# Supplementary material for: Effects of a novel mobile health intervention compared to a multi-component behaviour changing program on body mass index, physical capacities and stress parameters in adolescents with obesity: a randomized controlled trial
Source: BMC Pediatr. 2021 Jul 9;21:308. doi: 10.1186/s12887-021-02781-2 (PMC8266630; doi:10.1186/s12887-021-02781-2)
Supplement: Supplementary file 4 — Additional file 4. Table – Median values of fitness tests at each point time and change to intervention start (D1, D2). [file 12887_2021_2781_MOESM4_ESM.pdf]

Additional file 4 – Median values of fitness tests at each point time and change to intervention start (D1, D2).

			**T0**	**T1**	**T2**	**D1 (T1–baseline)**	**D2 (T2–baseline)**
**Side jumps (n/30 sec)**	PM	median (range)	71.5 (51 to 84)	81.5 (63 to 99)	84.0 (64 to 101)	12.0** (2 to 27)	14.0* (2 to 24)
	CON	median (range)	69.0 (62 to 82)	80.0 (67 to 103)	79.0 (70 to 114)	9.0* (4 to 23)	11.0* (3 to 32)
		p-value[a] (group differences)	0.79	0.58	0.61	0.4	0.41
**Tap Test (sec/25 cycles)**	PM	median (range)	12.4 (10 to 21)	10.6 (8 to 17)	10.9 (7 to 16)	-1.6** (-3 to 1)	-2.7* (-5 to -0)
	CON	median (range)	11.7 (10 to 16)	11.3 (9 to 14)	10.8 (8 to 15)	-1.4* (-3 to 1)	-1.3* (-2 to 0)
		p-value[a] (group differences)	0.49	0.88	0.93	0.24	0.055
**6-minute-run (km)**	PM	median (range)	0.75 (0.5 to 1.0)	0.79 (0.6 to 0.9)	0.81 (0.6 to 0.9)	0.03 (-0.1 to 0.2)	0.05 (-0.1 to 0.2)
	CON	median (range)	0.79 (0.7 to 0.9)	0.83 (0.7 to 1.1)	0.83 (0.7 to 1.1)	0.05* (-0.0 to 0.3)	0.12 (-0.1 to 0.2)
		p-value[a] (group differences)	0.2	0.14	0.21	0.4	0.28
**Balance (failures per min)**	PM	median (range)	2.0 (0 to 10)	0.0 (0 to 10)	0.0 (0 to 7)	0.0 (-4 to 7)	0.0 (-4 to 2)
	CON	median (range)	0.0 (0 to 3)	0.0 (0 to 2)	0.0 (0 to 1)	0.0 (-3 to 2)	0.0 (0 to 0)
		p-value[a] (group differences)	0.13	0.15	0.89	0.98	0.19

**Jump distance (cm)**	PM	median (range)	121.0 (100 to 171)	131.0 (97 to 189)	137.0 (103 to 182)	7.5* (-20 to 25)	11.0 (-21 to 29)
	CON	median (range)	127.0 (106 to 172)	142.0 (108 to 177)	145.0 (100 to 196)	10.5* (-19 to 35)	18.0 (-27 to 56)
		p-value[a] (group differences)	0.35	0.24	0.11	0.67	0.12
**Sit-ups (n/40 s)**	PM	median (range)	18.0 (2 to 26)	20.5 (3 to 28)	20.0 (12 to 30)	1.0* (-7 to 8)	4.5* (-1 to 11)
	CON	median (range)	19.0 (5 to 27)	23.5 (16 to 28)	23.0 (18 to 28)	2.5* (-1 to 11)	3.0* (-2 to 13)
		p-value[a] (group differences)	0.6	0.33	0.3	0.37	1
**Push-ups (n/ 40 s)**	PM	median (range)	8.5 (0 to 13)	9.5 (0 to 18)	10.5 (0 to 18)	1.5* (0 to 9)	3.0* (0 to 10)
	CON	median (range)	8.0 (4 to 17)	11.5 (6 to 18)	13.0 (10 to 19)	3.0* (0 to 6)	4.0* (1 to 7)
		p-value[a] (group differences)	0.42	0.35	0.2	0.8	0.57
**Sit and reach – Flexibility (cm)**	PM	median (range)	-3.5 (-16 to 9)	0.5 (-14 to 13)	-2.0 (-18 to 15)	4.0** (-1 to 12)	2.5 (-4 to 11)
	CON	median (range)	1.0 (-15 to 14)	0.0 (-16 to 13)	-3.0 (-16 to 13)	2.5 (-3 to 4)	0.0 (-3 to 7)
		p-value[a] (group differences)	0.42	0.9	1	0.035	0.51
Number of patients (all tests)	PM	n	18	18	14	18	14
	CON	n	13	12	9	12	9

* p-values <u><</u>0.05, ** p-values <0.001

[a] p-values testing the significance of group differences at each time point, and of group differences in change. Null hypothesis: medians of both groups are equal.

PM: PathMate group, CON: Control group, T0: intervention start, T1 and T2: 5.5 and 12 months after intervention start, respectively
