# Supplementary material for: Effects of a novel mobile health intervention compared to a multi-component behaviour changing program on body mass index, physical capacities and stress parameters in adolescents with obesity: a randomized controlled trial
Source: BMC Pediatr. 2021 Jul 9;21:308. doi: 10.1186/s12887-021-02781-2 (PMC8266630; doi:10.1186/s12887-021-02781-2)
Supplement: Supplementary file 5 — Additional file 5. Table – Median values of blood pressure and pulse at each point time and change to intervention start (D1, D2). [file 12887_2021_2781_MOESM5_ESM.pdf]

Additional file 5 – Median values of blood pressure and pulse at each point time and change to intervention start (D1, D2).

			**T0**	**T1**	**T2**	**D1 (change T1–T0)**	**D2 (change T2–T0)**
**Systolic blood pressure (mmHg)**	PM	median (range)	120.0 (107 to 140)	123.0 (93 to 136)	122.5 (103 to 136)	-3.0 (-27 to18)	3.0 (-17 to 20)
		n	17	18	16	17	15
	CON	median (range)	128.0 (112 to 140)	117.0 (108 to 136)	118.0 (105 to 130)	-5.0* (-24 to 8)	-4.5 (-20 to 7)
		n	13	13	10	13	10
		p-value[a] (group differences)	0.32	0.67	0.25	0.41	0.11
**Diastolic blood pressure (mmHg)**	PM	median (range)	62.0 (53 to 77)	59.5 (57 to 77)	70.0 (57 to 85)	-3.0 (-12 to 20)	7.0 (-8 to 28)
		n	17	18	16	17	15
	CON	median (range)	67.0 (53 to 82)	65.0 (54 to 78)	65.5 (53 to 85)	-1.0 (-17 to 11)	0.5 (-10 to 9)
		n	13	13	10	13	10
		p-value[a] (group differences)	0.19	0.42	0.63	0.95	0.16
**Pulse (per min)**	PM	median (range)	82.0 (62 to 108)	90.5 (59 to 121)	89.5 (61 to 120)	4.0 (-14 to 26)	0.0 (-23 to 40)
		n	17	18	16	17	15
	CON	median (range)	82.5 (56 to 124)	77.0 (55 to 92)	65.0 (51 to 104)	-1.0 (-34 to 11)	-7.0 (-38 to 33)
		n	12	12	10	11	9
		p-value[a] (group differences)	0.63	0.042	0.029	0.07	0.12

* p-values <0.05, ** p-values <0.001

[a] p-values testing the significance of group differences at each time point, and of group differences in change (null hypothesis: medians of both groups are equal)

PM: PathMate group, CON: Control group, T0: intervention start, T1 and T2: 5.5 and 12 months after intervention start, respectively
