# Supplementary material for: Effects of a novel mobile health intervention compared to a multi-component behaviour changing program on body mass index, physical capacities and stress parameters in adolescents with obesity: a randomized controlled trial
Source: BMC Pediatr. 2021 Jul 9;21:308. doi: 10.1186/s12887-021-02781-2 (PMC8266630; doi:10.1186/s12887-021-02781-2)
Supplement: Supplementary file 6 — Additional file 6. Table – Median values of TICS and cortisol at each time point and change to baseline (D1, D2). [file 12887_2021_2781_MOESM6_ESM.pdf]

Additional file 6 – Median values of TICS and cortisol at each time point and change to baseline (D1, D2).

			**Bl**	**T1**	**T2**	**D1 (change T1–Bl)**	**D2 (change T2–Bl)**
**TICS score (T-values, range 15-99)**	PM	median (range)	50.0 (37 to 59)	49.0 (35 to 66)	54.0 (37 to 65)	-2.0 (-11 to 16)	1.0 (-14 to 7)
		n	15	15	12	13	9
	CON	median (range)	58.5 (35 to 66)	56.0 (15 to 66)	53.0 (41 to 69)	-2.0 (-21 to 8)	3.5 (-25 to 6)
		n	12	11	8	11	8
		p-value[a] (group differences)	0.14	0.35	0.59	0.6	0.85
**Cortisol before relaxation in nmol/L**	PM	median (range)	220.0 (109 to 1307)	214.5 (98 to 402)	357.0 (122 to 576)	-11.0 (-1140 to 177)	85.5 (-1146 to 328)
		n	17	18	15	17	14
	CON	median (range)	219.5 (124 to 434)	239.0 (67 to 420)	312.0 (150 to 491)	-51.5 (-170 to 251)	78.0* (-19 to 311)
		n	12	13	9	12	8
		p-value[a] (group differences)	1	0.92	0.95	0.45	0.87
**Cortisol after relaxation (% change)**	PM	median (range)	-33.9[#] (-72 to 140)	-34.2[##] (-63 to 18)	-43.6 (-55 to 58)	0.9 (-172 to 67)	8.4 (-187 to 97)
		n	17	16	13	15	12
	CON	median (range)	-35.6[##] (-78 to -10)	-39.2[##] (-80 to 23)	-56.4[##] (-72 to -11)	-7.6 (-39 to 70)	-12.5 (-62 to 66)
		n	11	10	9	8	7
		p-value[a]	1	0.979	0.144	0.825	0.261

* p-values <0.05: Testing whether change from T0 to T1 or T2 within groups differs from 0.

[#] p-values <0.05, [##] p-values <0.001: Testing whether percentage of cortisol change after relaxation within groups at each point time differs from 0.

[a] p-values testing the significance of group differences at each time point, and of group differences in change. Null hypothesis: medians of both groups are equal.

Bl: baseline, on average 5 weeks before the start of intervention

PM: PathMate group, CON: Control group, T1 and T2: 5.5 and 12 months after intervention start, respectively

TICS: Trierer Stress Inventar questionnaire
Cortisol after relaxation (% change): percentage change of plasma cortisol after biofeedback relaxation exercise compared to cortisol before relaxation
