# Supplementary material for: Effects of a novel mobile health intervention compared to a multi-component behaviour changing program on body mass index, physical capacities and stress parameters in adolescents with obesity: a randomized controlled trial
Source: BMC Pediatr. 2021 Jul 9;21:308. doi: 10.1186/s12887-021-02781-2 (PMC8266630; doi:10.1186/s12887-021-02781-2)
Supplement: Supplementary file 7 — Additional file 7. App usage analytics – Table a. Smartphone app and PathMate2 app usage during the intervention period, i.e. 162 days (N = 11). Table b. Most-frequently used smartphone apps during the intervention period, i.e. 162 days (N = 11). [file 12887_2021_2781_MOESM7_ESM.pdf]

Additional file 7 – App usage analytics

Table a. Smartphone app and PathMate2 app usage during the intervention period, i.e. 162 days (N=11). Note: The duration was measured with the Android *App Usage Tracker* app by Vaibhav Agrawal.[1] Due to technical issues (e.g. the app background process was suspended by the Android operating system) or human involvement (e.g. the patients or parents stopped the app), data from 11 PM participants is reported.

Statistic	Total smartphone app usage per day in minutes	PathMate2 app usage per day in minutes	Ratio of PathMate2 app usage and total smartphone app usage in percent
Mean	16.2	0.8	16.0%
Median	6.0	0.3	3.9%
SD	26.8	1.5	0.2
Min	0.3	0.0	0.6%
Max	88.6	5.2	62.4%

Table b. Most-frequently used smartphone apps during the intervention period, i.e. 162 days (N=11). Note: App usage was measured with the Android *App Usage Tracker* app by Vaibhav Agrawal.[1] Due to technical issues (e.g. the app background process was suspended by the Android operating system) or human involvement (e.g. the patients or parents stopped the app), data from 11 PM participants is reported.

Rank	App	App Category	N	Freq in %
1	YouTube	Social Media	5	45.5%
2	PathMate2	Health & Fitness	3	27.3%
3	WhatsApp	Social Media	1	9.1%
3	SnapChat	Social Media	1	9.1%
3	Big Fish Casino	Mobile Game	1	9.1%

app: application, PM: PathMate group, SD: standard deviation

[1] https://play.google.com/store/apps/details?id=com.agrvaibhav.AppUsageTracking&hl=en
