# Supplementary material for: Effects of a novel mobile health intervention compared to a multi-component behaviour changing program on body mass index, physical capacities and stress parameters in adolescents with obesity: a randomized controlled trial
Source: BMC Pediatr. 2021 Jul 9;21:308. doi: 10.1186/s12887-021-02781-2 (PMC8266630; doi:10.1186/s12887-021-02781-2)
Supplement: Supplementary file 8 — Additional file 8. Figure – Associations between overall standardized fitness level and changes in BMI-SDS and body composition measures from T0 to T1. [file 12887_2021_2781_MOESM8_ESM.pdf]

Additional file 8 – Associations between overall standardized fitness level / changes in BMI-SDS and body composition measures from T0 to T1.

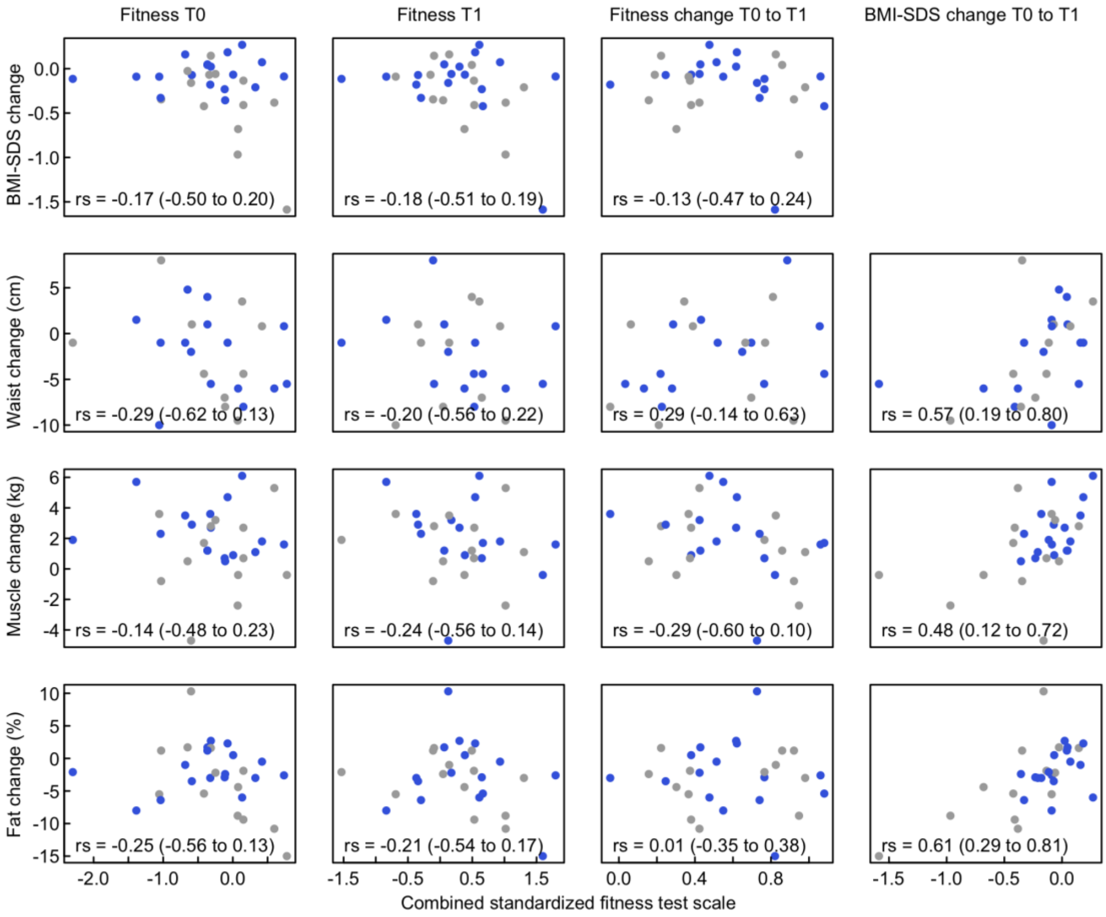

First three columns from left to right: Associations between overall standardized fitness level at T0, T1, change from T0 to T1 and changes in BMI-SDS, waist circumference, muscle mass and fat percentage, all from T0 to T1
Most right column: Associations between changes in BMI-SDS and those in other body measures from T0 to T1.
Associations are visualized by scatter plots and measured by Spearman rank correlations with 95% confidence intervals.

T0 = Intervention start, T1 = after 5.5 months, BMI-SDS: body mass index standard deviation score
Point colours indicate groups (blue = PathMate group, grey = control group)
Overall standardized fitness level: Correlation patterns (not shown) suggested that fitness tests were best summarized by combining all test results with the exception of balance and flexibility (sit and reach) tests, because most patients had the best score already at T0. To make those measures comparable, each of them was standardized to a mean of 0 and a standard deviation of 1 across the two assessments (T0 and T1). The “overall standardized fitness score” was then calculated as the mean of those standardized measures.
