# Supplementary material for: Effects of a novel mobile health intervention compared to a multi-component behaviour changing program on body mass index, physical capacities and stress parameters in adolescents with obesity: a randomized controlled trial
Source: BMC Pediatr. 2021 Jul 9;21:308. doi: 10.1186/s12887-021-02781-2 (PMC8266630; doi:10.1186/s12887-021-02781-2)
Supplement: Supplementary file 9 — Additional file 9. Figure – Associations between changes in TICS and changes in BMI-SDS and waist circumference from T0 to T2. [file 12887_2021_2781_MOESM9_ESM.pdf]

Correlation between delta BMI-SDS T0-T2 (x axis) & delta TICS T0-T2 (y axis)

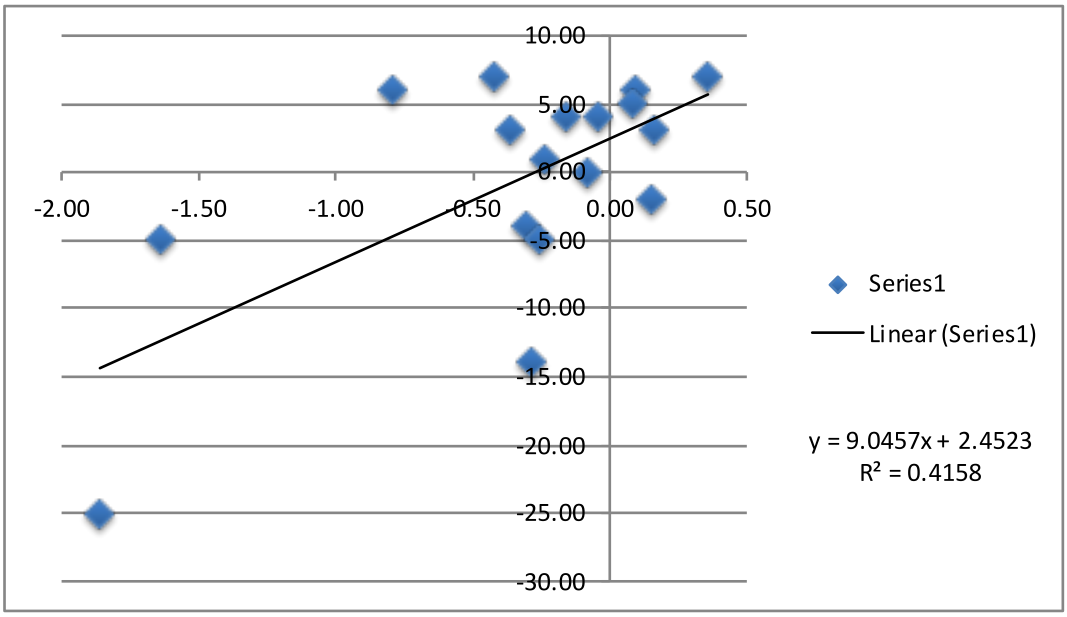

r = 0.64
r^2 = 0.42
n = 16
t_c = 0.23
t = 3.27, p = 0.005
Spearman's rho = 0.38, p = 0.13

Abbreviations

delta BMI-SDS T0-T2	BMI-SDS at T2 (12 months) minus BMI-SDS at T0 (start)
delta TICS T0-T2	TICS score at T2 (12 months) minus TICS score at T0 (start)
r	correlation coefficient
n	number of observations
t_c	t-test for the significance of correlation coefficient, t=r/SQRT((1-r^2)/(n-2))
SQRT	square root
Spearman's rho	Spearman's rank correlation coefficient
BMI-SDS	body mass index standard deviation score
TICS	Trierer Stress Inventar questionnaire

Correlation between delta TICS T0-T2 (x axis) & and delta WC T0-T2 (y axis)

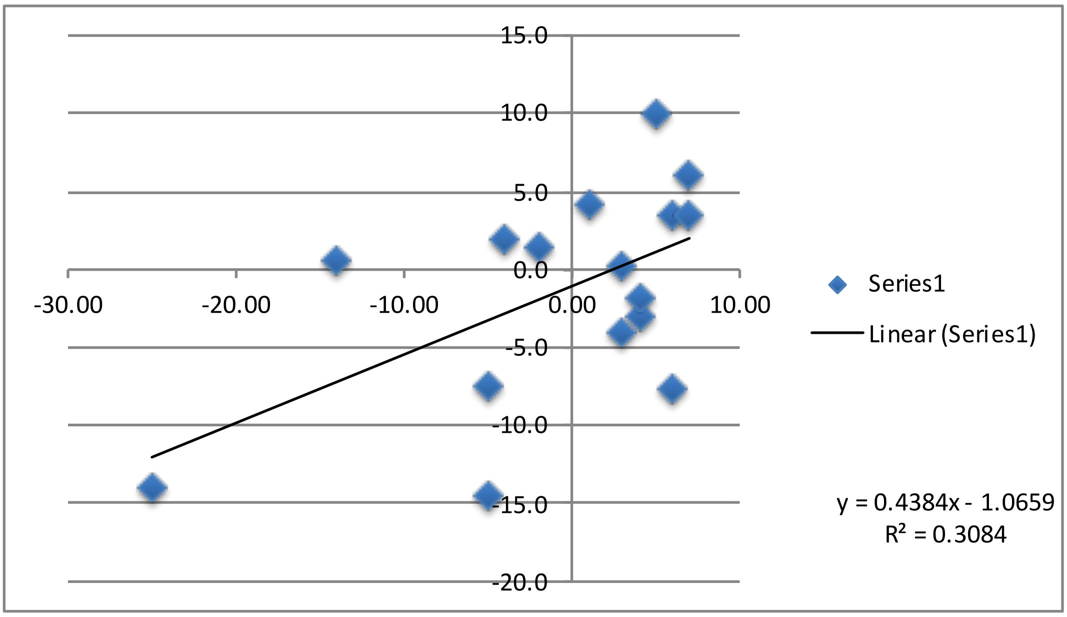

$r = 0.56$
$r^2 = 0.31$
$n = 16$
$t_c = 0.18$
$t = 2.50$, $p = 0.026$
Spearman's rho = 0.50, $p = 0.049$

Abbreviations

delta TICS T0-T2	TICS score at T2 (12 months) minus TICS score at T0 (start)
delta WC T0-T2	Waist circumference at T2 (12 months) minus waist circumference at T0 (start)
r	correlation coefficient
n	number of observations
t_c	t-test for the significance of correlation coefficient, t=r/SQRT((1-r^2)/(n-2))
SQRT	square root
Spearman's rho	Spearman's rank correlation coefficient
TICS	Trierer Stress Inventar questionnaire
