# Supplementary material for: Effects of a novel mobile health intervention compared to a multi-component behaviour changing program on body mass index, physical capacities and stress parameters in adolescents with obesity: a randomized controlled trial
Source: BMC Pediatr. 2021 Jul 9;21:308. doi: 10.1186/s12887-021-02781-2 (PMC8266630; doi:10.1186/s12887-021-02781-2)
Supplement: Supplementary file 11 — Additional file 11. Table – Analyses of group differences with generalized estimating equations (GEE) and calculations of intra- and intergroup effect sizes for the primary outcome and for physical capacities. [file 12887_2021_2781_MOESM11_ESM.pdf]

BMI-SDS (body mass index standard deviation score)

Test of effects based on a generalized estimating equations (GEE) model

Effect	X^2	p-value	
Time	9.6	0.008	0 vs. 5.5 vs. 12 months
Treatment group	2.0	0.160	
Time * group	6.6	0.036	

Estimation of effects based on this model	Coefficient	Wald test
Group difference in mean change from 0 to 5.5 months	0.335	p = 0.010
Group difference in mean change from 0 to 12 months	0.362	p = 0.096

Note: this effect is estimated on the original scale of measurement.

Estimation of intragroup and inter-group effect sizes using Cohen's d

These effects are estimated relative to to their standard deviation i.e. scale-independent.

Intragroup effect sizes

Group	Change	Cohen's d
PathMate (PM)	0 to 5.5 mo	-0.336
Control	0 to 5.5 mo	-0.842
PathMate	0 to 12 mo	-0.264
Control	0 to 12 mo	-0.539

Intergroup effect sizes calculated as the difference between intragroup effect sizes

PM vs. control	0 to 5.5 mo (D1)	0.506
PM vs. control	0 to 12 mo (D2)	0.275

Intergroup effect sizes calculated as Cohen's d for each time point

PM vs. control	0 months (T0)	0.170
PM vs. control	5.5 months (T1)	0.630
PM vs. control	12 months (T2)	0.512

Pooled standard deviation estimated from one-way analysis of variance.

Sidejump

Test of effects based on a generalized estimating equations (GEE) model

Effect	X^2	p-value	
Time	131.4	<0.001	0 vs. 5.5 vs. 12 months
Treatment group	0.0	0.850	
Time * group	0.4	0.810	

Estimation of effects based on this model	Coefficient	Wald test
Group difference in mean change from 0 to 5.5 months	1.47	0.519
Group difference in mean change from 0 to 12 months	0.99	0.744

Estimation of intragroup and inter-group effect sizes using Cohen's d

Intragroup effect sizes

Group	Change	Cohen's d
PathMate	0 to 5.5 mo	1.92

Control	0 to 5.5 mo	1.73
PathMate	0 to 12 mo	2.29
Control	0 to 12 mo	1.64

Intergroup effect sizes calculated as the difference between intragroup effect sizes

PM vs. control	0 to 5.5 mo (D1)	0.19
PM vs. control	0 to 12 mo (D2)	0.65

Intergroup effect sizes calculated as Cohen's d for each time point

PM vs. control	0 months (T0)	0.170
PM vs. control	5.5 months (T1)	0.630
PM vs. control	12 months (T2)	0.512

Tap test (time)

Test of effects based on a generalized estimating equations (GEE) model

Effect	X^2	p-value	
Time	69.9	<0.001	0 vs. 5.5 vs. 12 months
Treatment group	0.1	0.813	
Time * group	7.3	0.026	

Estimation of effects based on this model	Coefficient	Wald test
Group difference in mean change from 0 to 5.5 months	-0.52	0.178
Group difference in mean change from 0 to 12 months	-1.13	0.007

Estimation of intragroup and inter-group effect sizes using Cohen's d

Intragroup effect sizes

Group	Change	Cohen's d
PathMate	0 to 5.5 mo	-1.413
Control	0 to 5.5 mo	-1.023
PathMate	0 to 12 mo	-1.742
Control	0 to 12 mo	-1.493

Intergroup effect sizes calculated as the difference between intragroup effect sizes

PM vs. control	0 to 5.5 mo (D1)	-0.39
PM vs. control	0 to 12 mo (D2)	-0.249

Intergroup effect sizes calculated as Cohen's d for each time point

PM vs. control	0 months (T0)	0.283
PM vs. control	5.5 months (T1)	0.117
PM vs. control	12 months (T2)	-0.104

Run

Test of effects based on a generalized estimating equations (GEE) model

Effect	X^2	p-value	
Time	10.9	0.004	0 vs. 5.5 vs. 12 months
Treatment group	3.6	0.059	
Time * group	2.2	0.339	

**Estimation of effects based on this model**	**Coefficient**	**Wald test**
Group difference in mean change from 0 to 5.5 months	-0.047	0.168
Group difference in mean change from 0 to 12 months	-0.055	0.198

Estimation of intragroup and inter-group effect sizes using Cohen's d

Intragroup effect sizes

**Group**	**Change**	**Cohen's d**
PathMate	0 to 5.5 mo	0.385
Control	0 to 5.5 mo	0.723
PathMate	0 to 12 mo	0.448
Control	0 to 12 mo	0.65

Intergroup effect sizes calculated as the difference between intragroup effect sizes

PM vs. control	0 to 5.5 mo (D1)	-0.338
PM vs. control	0 to 12 mo (D2)	-0.202

Intergroup effect sizes calculated as Cohen's d for each time point

PM vs. control	0 months (T0)	-0.362
PM vs. control	5.5 months (T1)	-0.767
PM vs. control	12 months (T2)	-0.656

One leg balance (failures, square-root transformed)

Test of effects based on a generalized estimating equations (GEE) model

**Effect**	X^2	**p-value**	
Time	4.8	0.090	0 vs. 5.5 vs. 12 months
Treatment group	3.1	0.078	
Time * group	5	0.080	

**Estimation of effects based on this model**	**Coefficient**	**Wald test**
Group difference in mean change from 0 to 5.5 months	0.022	0.948
Group difference in mean change from 0 to 12 months	-0.414	0.693

Estimation of intragroup and inter-group effect sizes using Cohen's d

Intragroup effect sizes

**Group**	**Change**	**Cohen's d**	
PathMate	0 to 5.5 mo	-0.304	Note: Given the data distribution (many zeroes), this analysis is not meaningful.
Control	0 to 5.5 mo	-0.304	
PathMate	0 to 12 mo	-0.5	
Control	0 to 12 mo		Calculation not possible because the standard deviation is 0.

Intergroup effect sizes calculated as the difference between intragroup effect sizes

PM vs. control	0 to 5.5 mo (D1)	0
PM vs. control	0 to 12 mo (D2)	

Intergroup effect sizes calculated as Cohen's d for each time point

PM vs. control	0 months (T0)	0.575
PM vs. control	5.5 months (T1)	0.629
PM vs. control	12 months (T2)	0.204

Jump distance

Test of effects based on a generalized estimating equations (GEE) model

Effect	X^2	p-value	
Time	15.4	0.005	0 vs. 5.5 vs. 12 months
Treatment group	1.9	0.167	
Time * group	2.4	0.301	

Estimation of effects based on this model	Coefficient	Wald test
Group difference in mean change from 0 to 5.5 months	-2.826	0.563
Group difference in mean change from 0 to 12 months	-9.391	0.194

Estimation of intragroup and inter-group effect sizes using Cohen's d

Intragroup effect sizes

Group	Change	Cohen's d
PathMate	0 to 5.5 mo	0.656
Control	0 to 5.5 mo	0.691
PathMate	0 to 12 mo	0.622
Control	0 to 12 mo	0.84

Intergroup effect sizes calculated as the difference between intragroup effect sizes

PM vs. control	0 to 5.5 mo (D1)	-0.035
PM vs. control	0 to 12 mo (D2)	-0.218

Intergroup effect sizes calculated as Cohen's d for each time point

PM vs. control	0 months (T0)	-0.385
PM vs. control	5.5 months (T1)	-0.503
PM vs. control	12 months (T2)	-0.727

Situps

Test of effects based on a generalized estimating equations (GEE) model

Effect	X^2	p-value	
Time	28.2	<0.001	0 vs. 5.5 vs. 12 months
Treatment group	0.5	0.480	
Time * group	3.5	0.170	

Estimation of effects based on this model	Coefficient	Wald test
Group difference in mean change from 0 to 5.5 months	-1.475	0.267
Group difference in mean change from 0 to 12 months	-0.344	0.836

Estimation of intragroup and inter-group effect sizes using Cohen's d

Intragroup effect sizes

Group	Change	Cohen's d
PathMate	0 to 5.5 mo	0.522
Control	0 to 5.5 mo	0.983
PathMate	0 to 12 mo	0.978
Control	0 to 12 mo	0.963

Intergroup effect sizes calculated as the difference between intragroup effect sizes

PM vs. control	0 to 5.5 mo (D1)	-0.461
PM vs. control	0 to 12 mo (D2)	0.015

Intergroup effect sizes calculated as Cohen's d for each time point

PM vs. control	0 months (T0)	-0.131
PM vs. control	5.5 months (T1)	-0.489
PM vs. control	12 months (T2)	-0.504

Pushup

Test of effects based on a generalized estimating equations (GEE) model

Effect	X^2	p-value	
Time	46.6	<0.001	0 vs. 5.5 vs. 12 months
Treatment group	1.7	0.190	
Time * group	0.2	0.920	

Estimation of effects based on this model	Coefficient	Wald test
Group difference in mean change from 0 to 5.5 months	0.165	0.852
Group difference in mean change from 0 to 12 months	-0.089	0.932

Estimation of intragroup and inter-group effect sizes using Cohen's d

Intragroup effect sizes

Group	Change	Cohen's d
PathMate	0 to 5.5 mo	0.867
Control	0 to 5.5 mo	1.196
PathMate	0 to 12 mo	1.069
Control	0 to 12 mo	1.969

Intergroup effect sizes calculated as the difference between intragroup effect sizes

PM vs. control	0 to 5.5 mo (D1)	-0.329
PM vs. control	0 to 12 mo (D2)	-0.9

Intergroup effect sizes calculated as Cohen's d for each time point

PM vs. control	0 months (T0)	-0.444
PM vs. control	5.5 months (T1)	-0.370
PM vs. control	12 months (T2)	-0.522

Reach flexibility

Test of effects based on a generalized estimating equations (GEE) model

Effect	X^2	p-value	
Time	27.2	<0.001	0 vs. 5.5 vs. 12 months
Treatment group	0.2	0.630	
Time * group	8.7	0.010	

Estimation of effects based on this model	Coefficient	Wald test
Group difference in mean change from 0 to 5.5 months	2.9	0.006
Group difference in mean change from 0 to 12 months	1.25	0.437

Estimation of intragroup and inter-group effect sizes using Cohen's d

Intragroup effect sizes

Group	Change	Cohen's d
PathMate	0 to 5.5 mo	1.261
Control	0 to 5.5 mo	0.608
PathMate	0 to 12 mo	0.542
Control	0 to 12 mo	0.295

Intergroup effect sizes calculated as the difference between intragroup effect sizes

PM vs. control	0 to 5.5 mo (D1)	0.653
PM vs. control	0 to 12 mo (D2)	0.247

Intergroup effect sizes calculated as Cohen's d for each time point

PM vs. control	0 months (T0)	-0.340
PM vs. control	5.5 months (T1)	0.130
PM vs. control	12 months (T2)	-0.008
